# A *tti1* mutation in the Tel2-Tti1-Tti2 complex specifically eliminates the cellular function of Rad3^ATR^, but not that of other PIKKs in fission yeast

**DOI:** 10.1371/journal.pgen.1012206

**Published:** 2026-06-11

**Authors:** Sankhadip Bhadra, Nafees Ahamad, Saman Khan, Yong-jie Xu

**Affiliations:** Department of Pharmacology and Toxicology, Boonshoft School of Medicine, Wright State University, Dayton, Ohio, United States of America; Geisel School of Medicine at Dartmouth, UNITED STATES OF AMERICA

## Abstract

The Tel2-Tti1-Tti2, or TTT complex, is the co-chaperone for co-translational maturation of all phosphatidylinositol 3-kinase-related kinases (PIKKs). The complex is highly conserved in eukaryotes and controls multiple cellular processes through PIKKs. Mutations of the TTT complex have recently been linked to disease syndromes and cancer. In *Schizosaccharomyces pombe*, six PIKKs are expressed: Rad3^ATR^, Tel1^ATM^, Tor1 and Tor2 (homologs of mTOR), and Tra1 and Tra2 (homologs of TRRAP). While Rad3^ATR^ and Tel1^ATM^ are the central cellcycle checkpoint kinases in response to DNA damage and replication stress, the other four PIKKs govern cell growth, nutrient sensing, and transcriptional regulation. Here, we report the identification of seven *tti1* mutants in fission yeast that are sensitive to genotoxins. Characterization of one of the mutants, *tti1-N18*, reveals that the mutation selectively eliminates the kinase function of Rad3^ATR^, but not that of Tel1^ATM^. Further examination shows that, like Tel1^ATM^, the functions of the other four PIKKs are also largely uncompromised in the *tti1-N18* mutant. These findings suggest a mechanism by which the TTT complex confers functional specificity towards Rad3^ATR^ among the PIKKs. Since human Tel2 has been identified as a target of the antiparasitic drug Ivermectin, further investigation of the substrate specificity of the TTT complex may reveal a therapeutic vulnerability for treatment of cancer or other diseases.

## Author summary

Phosphatidylinositol 3-kinase-related kinases, or PIKKs, are a group of large kinases that are structurally similar to lipid kinases but have protein kinase activities. Although only six PIKKs are expressed in mammalian cells, they are crucial for genome maintenance and cell growth. Before PIKKs become active, a Tel2-Tti1-Tti2 (TTT) complex is necessary for their co-translational maturation. Therefore, TTT regulates multiple cellular processes through PIKKs. Consistent with its biological importance, TTT is highly conserved in eukaryotes, like PIKKs. While studying the potential role of TTT in regulating the DNA replication checkpoint in fission yeast, we identified a mutant, *tti1-N18*, which has a moderately destabilized TTT. Surprisingly, although the checkpoint function of Rad3, the ortholog of ATR and one of the six mammalian PIKKs, is lost in *tti1-N18*, the functions of other PIKKs remain largely intact. We propose that TTT may specifically recognize each newly synthesized PIKK for co-translational maturation. Since TTT has been confirmed as a therapeutic target, further investigation into the substrate specificity may enable targeting a specific set of PIKKs for cancer treatment.

## Introduction

PIKKs are a group of large protein kinases of 270–450 kDa [1–3]. Mammalian cells express six PIKKs: ATM (ataxia-telangiectasia mutated), ATR (ATM and Rad3-related protein), DNA-PKcs (the catalytic subunit of DNA-dependent protein kinase), mTOR (mammalian target of rapamycin), TRRAP (transformation/transcription domain-associated protein), and SMG-1(suppressor with morphogenetic effect on genitalia 1). The PIKKs mediate diverse biological functions through the conserved lipid kinase-like protein kinase domain located near the C-terminus that phosphorylates serine and threonine residues on target proteins. The kinase domain, accounting for only 5–10% of the total sequence, is flanked by the conserved FAT (FRAP-ATM-TRRAP) and FATC (FAT C-terminus) domains. Most of the N-termini of the PIKKs are composed of long arrays of HEAT (huntingtin, elongation factor 3, protein phosphatase 2A, and TOR1) repeats [4]. Although TRRAP is catalytically inactive, it shares an overall structure with other PIKKs [3].

The PIKKs represent only a small fraction of the human kinome [5], but they regulate diverse cellular processes crucial for cell growth and proliferation. ATM and ATR are conserved in all eukaryotes. They govern cellular responses to DNA damage or replication stress by phosphorylating proteins involved in DNA repair and cell cycle regulation [6]. Although partially redundant, ATM is activated in response to DNA double-strand breaks (DSBs), and ATR is activated by single-strand DNA (ssDNA) generated at the DNA damage sites or stalled replication forks. DNA-PKcs is crucial for DNA repair of DSBs by nonhomologous end-joining [7]. SMG1 regulates nonsense-mediated decay of aberrant mRNA [8]. mTOR is the catalytic subunit of TORC1 and TORC2 complexes that control cell growth in response to nutrient availability, mitogenic signals, and environmental cues [9]. The catalytically inactive TRRAP regulates gene expression through association with histone acetyltransferase complexes [10]. Remarkably, all these important biological functions of the six PIKKs are controlled by the TTT complex [11,12].

The TTT complex was discovered in the model organisms of *C. elegans* and yeasts over the past four or five decades [12]. Like PIKKs, the TTT is highly conserved in eukaryotes. All three subunits of the complex are essential for cell survival. The TTT interacts with the RUVBL1-RUVBL2-Tah1-Pih1, or R2TP complex, and other proteins. It acts as a molecular adaptor that recognizes newly synthesized PIKKs and functions as a co-chaperone of Hsp90 for the co-translational maturation of PIKKs [11,13,14]. The cryo-EM structures of the TTT revealed that the three subunits are largely composed of HEAT repeats that are elongated, helical in nature and folded into an ɑ-solenoid structure [15,16]. They stabilize one another via specific binding interfaces in the complex. A recent study in the fission yeast *S. pombe* shows that during ribosomal translation, the TTT does not bind to the newly translated HEAT repeats of the PIKKs [17]. Once the more conserved FAT and kinase domains, or FATKIN, are translated, the TTT binds to the FATKIN to shield the premature PIKKs from binding to their respective partners, and, in collaboration with Hsp90 and the R2TP complex, assists the co-translational maturation of all PIKKs [17]. The FATC domain of the PIKKs, particularly the last two hydrophobic residues, also plays a crucial role in the maturation process [17]. Although poorly understood, the properly folded PIKKs are expected to associate with their respective binding partners to mediate the biological functions described above [12].

TTT is essential for cell growth. However, non-lethal mutations of the complex have been reported in multiple model organisms [12]. These mutants exhibit pleiotropic phenotypes consistent with defects in multiple cellular processes regulated by the PIKKs. In humans, non-lethal mutations of the TTT complex have been linked to diseases, such as You-Hoover-Fong syndrome [18–21], characterized by intellectual disability, global developmental delay, microcephaly, and abnormal auditory and visual functions. Recent studies have also linked TTT activities or mutations with various types of cancer [22,23], suggesting that it can serve as an important therapeutic target. In support of this notion, human Tel2 has been identified as a target of Ivermectin [24,25], an antiparasitic drug that has treated millions of patients with river blindness, elephantiasis, and scabies. This discovery validates the TTT as a druggable target for the treatment of cancer or other diseases.

The ATR homolog in the fission yeast *S. pombe* is Rad3. Like ATR, Rad3 is the master kinase of the DNA replication checkpoint (DRC) and the DNA damage checkpoint (DDC) pathways [26]. Unlike ATM in mammalian cells, Tel1, the homolog of ATM, contributes minimally to the checkpoints in fission yeast. While studying the mechanisms of the DRC, we have screened a group of mutants that are sensitive to hydroxyurea (HU), an inducer of replication stress [27]. Among the screened mutants, the *tel2-C307Y* mutation in the TTT has been reported to eliminate Rad3 kinase signaling in the DRC but moderately reduces the Rad3 kinase signaling in the DDC pathway [28]. This suggests that the TTT may regulate the DRC downstream of Rad3 in fission yeast.

To better understand the TTT and its potential role in the DRC, we employed a targeted forward genetic approach to screen non-lethal mutants of *tti1*, which encodes the largest subunit of the TTT. Here we report the identification of seven *tti1* mutants that are highly sensitive to HU, methyl methane sulfonate (MMS), and other genotoxins. Consistent with the drug sensitivities, we show that, like the *tel2-C307Y* mutant [28], the *tti1* mutations moderately reduced the protein levels of Rad3^ATR^. Surprisingly, while the Rad3^ATR^ function is abolished in one of the *tti1* mutants, *tti1-N18*, the protein level and the kinase activity of Tel1^ATM^ remain unaffected. We then examined the other PIKKs in fission yeast and found that the protein levels and the cellular functions of other PIKKs are largely uncompromised in the *tti1-N18* mutant. We propose that the co-translational maturation of PIKKs mediated by the TTT involves substrate-specific recognition. Although other possibilities remain, further investigation of such specificity may provide a therapeutic strategy to target a particular set of PIKK pathways.

## Results

### Identification of seven *tti1* mutants that are sensitive to HU and MMS

**Fig 1:**
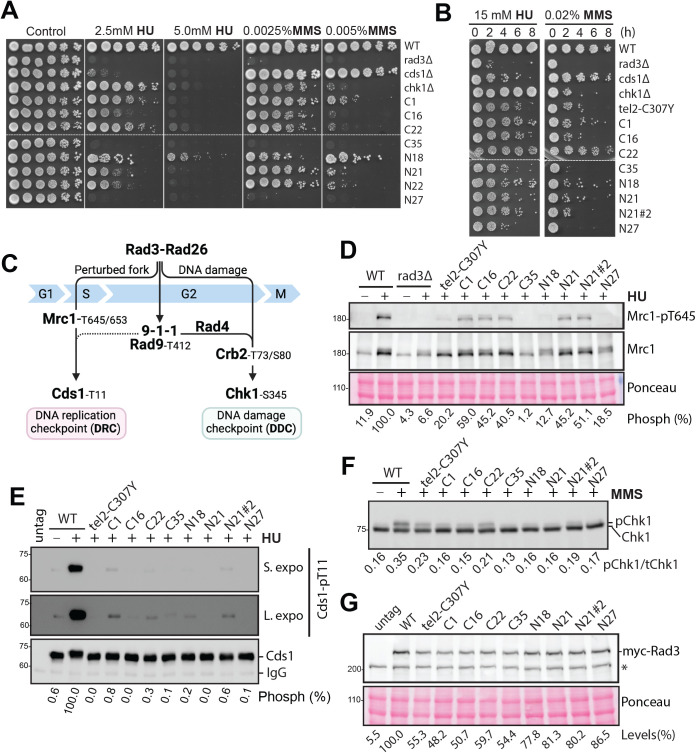
Identification of seven *tti1* mutants with defective Rad3^ATR^ kinase signaling in the checkpoint pathways. **(A)** Sensitivities of the screened *tti1* mutants to HU and MMS were examined by spot assay. Serial five-fold dilutions of logarithmically growing cells were spotted on YE6S plates or YE6S plates containing HU or MMS at the indicated concentrations. The plates were incubated at 30˚C for 3 days and then photographed. Wild-type *S. pombe* and the checkpoint mutants *rad3∆*, *cds1∆*, and *chk1∆* were included as controls. The dashed line indicates discontinuity. **(B)** Sensitivities of the *tti1* mutants to acute treatment with HU or MMS were determined by spot assay. Logarithmically growing cells were treated with 15 mM HU or 0.02% MMS in liquid cultures. Equal amounts of the cultures were removed at the indicated time points, washed once, diluted fivefold, and then spotted on YE6S plates for cell recovery at 30˚C for 3 days. Note: because *N22* has the identical mutations as in *N21*, it was renamed *N21#2*. Dashed lines indicate discontinuity. **(C)** Diagram of Rad3^ATR^ (with its cofactor Rad26^ATRIP^)-mediated phospho-signaling in the DNA replication (DRC, left) and DNA damage checkpoint (DDC, right) pathways in fission yeast. Numbers indicate Rad3-specific phosphorylation sites. Rad3 phosphorylates Mrc1 and Cds1 to activate the DRC [33,34]. In the presence of DNA damage, it phosphorylates Crb2 and Chk1 to activate the DDC [36–38]. Rad3 phosphorylation of Rad9 in the 9-1-1 checkpoint clamp is required to activate the DRC and the DDC [34,40], although the exact function of the phosphorylation in the DRC remains undefined (dashed line). **(D)** Rad3 phosphorylation of Mrc1-T645 was reduced or eliminated in the *tti1* mutants. Wild-type *S. pombe* and the mutants with the indicated mutations were treated with 15 mM HU for 3 **h.** Mrc1-pT645 (upper panel) was detected in whole-cell lysates using a phospho-specific antibody. The blot was stripped, washed extensively, and reprobed with anti-Mrc1 antibodies (middle panel). A section of the Ponceau S-stained membrane is shown as the loading control (bottom panel). The Mrc1-pT645 bands were quantified and shown at the bottom relative to HU-treated wild-type cells. **(E)** Phosphorylation of Cds1-T11 by Rad3 was eliminated in the *tti1* mutants. Wild-type *S. pombe*, *tel2-C307Y*, and *tti1* mutant cells were treated with 15 mM HU for 3 **h.** Cds1 was IPed and then analyzed by Western blotting using anti-HA antibody (bottom panel). The same membrane was stripped, washed, and reprobed with the phospho-specific antibody against Cds1-pT11 (upper two panels). The phosphorylation bands were quantified, and the relative intensities are shown at the bottom. **(F)** Rad3 phosphorylation of Chk1 in the DDC pathway was examined by mobility shift assay in wild-type, *tel2-C307Y,* and the *tti1* mutant cells treated with 0.001% MMS for 90 min. Whole-cell lysates were analyzed by SDS-PAGE followed by Western blotting using anti-myc antibody. **(G)** Protein levels of Rad3 were examined by Western blotting using anti-myc antibody in wild-type *S. pombe*, *tel2-C307Y,* and the *tti1* mutants under physiological conditions. The asterisk indicates a cross-reactive material. A section of the Ponceau S-stained membrane is shown as a loading control (bottom panel). The intensities of the Rad3 bands were quantified and shown in percentages at the bottom.

To investigate the potential role of the TTT in checkpoint regulation, we screened for non-lethal *tti1* mutants that are sensitive to HU and MMS using the targeted forward genetic approach described previously [29,30]. Since *tti1* is a large gene, we engineered a silent mutation in the middle of the gene body to generate an NheI site (S1 Fig A). The *tti1* expression cassette was cloned into the pJK210 integration vector carrying a *ura4* marker [31] (S1 Fig B). Random mutations were generated by mutational PCRs [32] in the N- and C-terminal halves of *tti1* in two separate libraries. Allele replacement at the *tti1* genomic locus was achieved by transforming wild-type *S. pombe* lacking the *ura4* gene with linearized library DNA. The transformed cells with non-lethal mutations were selected using the pop-in and the pop-out method. Colonies were replicated onto YE6S plates containing HU or MMS to select sensitive colonies. The drug-sensitive colonies were streaked out to single colonies and their drug sensitivities were confirmed by spot assay. Some of the representative mutants are shown in S1 Fig C. The primary mutants were backcrossed at least once before DNA sequencing to identify the mutations in *tti1*. After removing redundant and non*-tti1* mutations, the mutants were renamed. In total, seven *tti1* mutants with different mutations were screened (Fig 1A). Among them, *C35* and *N27* showed the highest drug sensitivity, comparable to *S. pombe* lacking Rad3^ATR^. The mutations identified by Sanger sequencing in the seven *tti1* mutants are shown in S2 Fig and summarized in Table 1. The mutated residues, their distribution in the primary sequence, and the conservation among yeasts and humans are highlighted in S3 Fig. Since *N22* shares the same mutations as in *N21*, we refer to *N22* as *N21#2* mutant in the following experiments. We also examined the drug sensitivity of the screened *tti1* mutants in liquid cultures, which confirmed their sensitivities to acute drug treatment (Fig 1B).

**Table 1:** The identified missense mutations in the seven *tti1* mutants.

tti1 mutants	Amino acid substitutions in Tti1
*C1*	S625L-E759V-E840D-R910K-L989M-L1058T-S1076P
*C16*	A704T-H758Y-W874R-E1008K-G1028R
*C22*	A613V-L646F-I663M-V678A-A692T-N728S-C735S-V742A-R912H-A932T-E1071*
*C35*	D667G-M811K-V823I-F906S-H927Y-T993A-A996T-T1035I-H1040R
*N18*	P70Q-I367V-G457D
*N21*	A44E-T110S-K249N-I317V-V387D-M420V
*N27*	L25Q-P70L-I367F-Q194L-E466V

Note: The silent mutations are not shown. E1071* in C22 denotes a nonsense mutation.

### Defective Rad3^ATR^ kinase signaling in the *tti1* mutants

Rad3, together with its cofactor Rad26^ATRIP^, is the master sensor kinase of the DRC and DDC pathways in fission yeast (Fig 1C). In the DRC pathway, Rad3 phosphorylates two TQ motifs (T645 and T653) in the middle of Mrc1, the mediator protein of the DRC [33,34]. Phosphorylated Mrc1 recruits the effector kinase Cds1^CHK2^ to be phosphorylated by Rad3 at Cds1-T11. Phosphorylated Cds1-T11 promotes homodimerization and autophosphorylation of Cds1-T328 [35]. Phosphorylation of Cds1-T328 directly activates Cds1 [35], which mediates most of the biological functions of the DRC. When DNA damage occurs at G2, the longest cell cycle time in fission yeast, Rad3 phosphorylates Chk1-S345 to activate the DDC [36–38]. Like Cds1, activated Chk1 mediates most of the biological functions of the DDC pathway.

To investigate the potential DRC defects in the *tti1* mutants, we treated wild-type and mutant *S. pombe* with 15 mM HU for 3 h. Rad3 phosphorylation of Mrc1 was examined in whole cell lysates using a phosphor-specific antibody against Mrc1-pT645 (Fig 1D, top panel). In the presence of HU, Mrc1 phosphorylation was significantly increased in wild-type cells. Since Mrc1 is specifically expressed at G1/S and activated DRC promotes its expression [33,39], HU treatment also increased the protein level of Mrc1 (Fig 1D, middle panel). Because Rad3 specifically phosphorylates Mrc1-T645, the phosphorylation was not detected in cells lacking Rad3. As previously reported, the *tel2-C307Y* mutation almost eliminated Mrc1-T645 phosphorylation [28]. Under similar conditions, we found that, while *C1*, *C16*, *C22*, and *N21* mutants showed a significantly reduced phosphorylation, Mrc1 phosphorylation was eliminated in *C35*, *N18*, and *N27* mutants. We then examined Rad3 phosphorylation of Cds1 in the presence of HU and found that, like the *tel2-C307Y* mutant, Cds1 phosphorylation was almost eliminated in the *tti1* mutants (Fig 1E). These results showed a significant defect in Rad3 kinase signaling at the HU-treated fork in *tel2-C307Y* and the newly screened *tti1* mutants.

We next examined Rad3 phosphorylation of Chk1 in the presence of MMS by mobility shift assay (Fig 1F). As previously reported, the phosphorylation of Chk1 was moderately decreased in the *tel2-C307Y* mutant [28]. Under similar conditions, we found that while *C35*, *N18*, and *N27* mutations reduced phosphorylation to background level, like untreated wild-type cells, *C1*, *C16*, *C22*, and *N21* mutants showed moderately reduced Chk1 phosphorylation. Since the *tel2-C307Y* mutation moderately reduces Rad3 protein level [28], we examined Rad3 protein levels in the *tti1* mutants. Western blotting showed that Rad3 levels were moderately reduced in all seven *tti1* mutants, like the *tel2-C307Y* mutant (Fig 1G). Together, we found that while the Rad3 level was moderately reduced in the *tti1* mutants, Rad3 phosphorylation of Mrc1, Cds1, and Chk1 was significantly compromised or eliminated, leading to drug sensitivities observed in Fig 1A and 1B.

### The mutations in *tti1* directly cause the checkpoint defects

Although it minimally contributes to the checkpoints, Tel1^ATM^ also phosphorylates Mrc1 in the presence of HU [33], which causes a mobility shift of Mrc1 in HU-treated *rad3∆* cells (Fig 1D, middle panel). Unlike *rad3∆* cells in which the Tel1 phosphorylation-induced Mrc1 mobility shift was observed, the mobility shift was not observed in the *C35* mutant, suggesting that the kinase functions of both Rad3 and Tel1 were eliminated. In contrast, while the phosphorylation of Mrc1-T645 was eliminated in *N18* and *N27* mutants, the mobility shift of Mrc1 remained unaffected, like in *rad3∆* or wild-type cells. This suggests that the kinase function of Tel1 was likely unaffected in the two mutants. Since *N18* and *N27* showed a similar defect in Mrc1 phosphorylation and shared two mutated residues, P70 and I367, in *tti1*, we further investigated the *tti1* mutants by focusing on *N18*, as it has fewer mutations than *N27*.

Although our screening approach targets the *tti1* genomic locus, we were concerned whether the screened *tti1* mutants carry off-target mutations in the genome that cause drug sensitivities and checkpoint defects. This is a valid concern because we also screened a few drug-sensitive mutants without the *tti1* mutation. To confirm that the identified *tti1* mutations in *N18* and the six other mutants directly cause the drug sensitivities, three different experiments were conducted: (1) tetrad dissection, (2) rescuing the mutants by expression of wild-type *tti1* on a vector, and (3) integration of the identified *N18* mutation at the *tti1* genomic locus in a wild-type strain.

**Fig 2:**
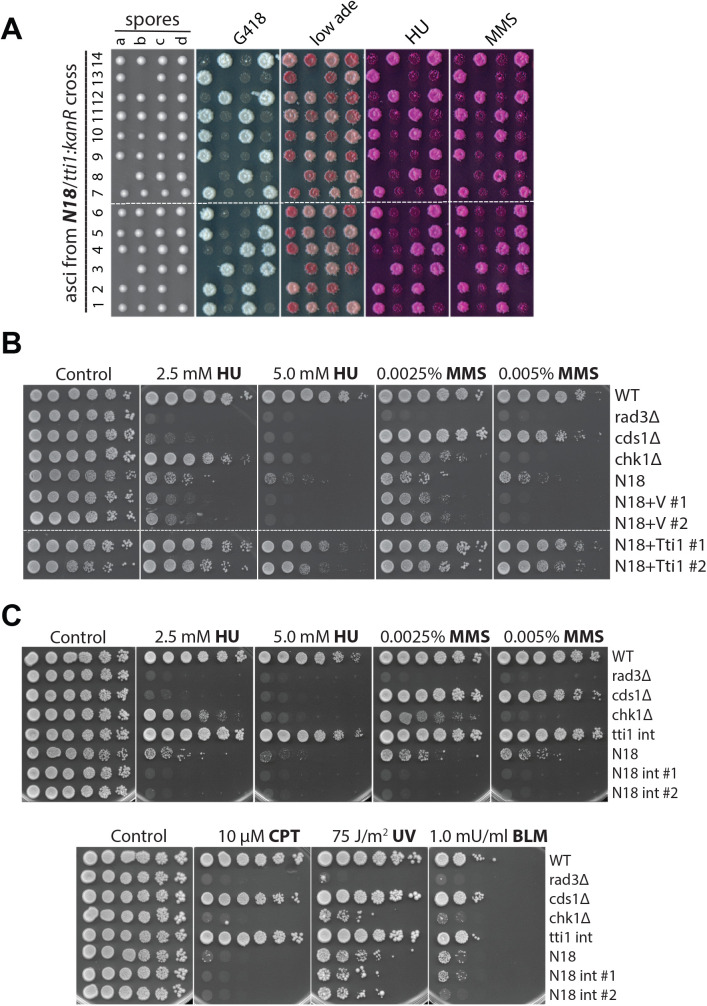
The *tti1* mutations cause drug sensitivities in the *N18* mutant. **(A)** Tetrad dissection of the asci from the cross between *N18* and the wild-type *S. pombe* in which *tti1* is linked with the *kanR* marker. The colonies formed from germinated spores were replicated onto a G418 plate to reveal the *kanR* marker, a low adenine plate for the two alleles of *ade6*, and HU and MMS plates containing the lethality dye phloxin B to reveal colonies with the *N18* mutation. All tetrads displayed 2:2 ratios on replica plates, and the absolute segregation of the *N18* mutation with the *tti1-*linked *kanR* marker. **(B)** Extra-chromosomal expression of wild-type Tti1 rescued the *N18* mutant. The *N18* mutant was transformed with an empty vector or the vector expressing *tti1* under its native promoter. Drug sensitivities were determined by spot assay as in Fig 1A. Two separate colonies from the vector control and the *tti1*-expression vector were examined. The dashed line indicates discontinuity. **(C)** The *N18* mutation was integrated at the *tti1* genomic locus, as illustrated in S5 Fig **B.** As a control, wild-type *tti1* was integrated by the same methods. The drug sensitivities were examined by spot assay. Two separate colonies were tested for the sensitivities to HU and MMS (top panels), and UV, camptothecin, and bleomycin at the indicated concentrations (lower panels). Compared with the primary mutant, the slightly increased drug sensitivities in the *N18* integrant are likely due to the C-terminal epitope tagging.

For the tetrad analysis, *N18* (Fig 2A) and other *tti1* mutants (S4 Fig) were crossed with a wild-type strain in which *tti1* is linked with a *kanR* marker. After dissection of the tetrads, the colonies formed on YE6S were replicated onto YE6S plates containing G418 to identify the *kanR* colonies or low adenine to reveal two different alleles of *ade6*. Red colonies carry the *ade6-M210* allele, while pink colonies express the *ade6-M216* allele. The expected 2:2 ratios of *kanR* and a*de6* alleles in all tetrads confirmed the successful dissection. The colonies were also replicated onto HU and MMS plates to identify the colonies with the *tti1* mutation. The results clearly showed that the *tti1* mutations were always segregated from *tti1:kanR*, which confirmed the *tti1* mutations in *N18* and the rest of the *tti1* mutants. We then expressed wild-type *tti1* from a vector under the control of its native promoter and found that the *N18* (Fig 2B) and the other six *tti1* mutants (S5 Fig A) were fully rescued from the drug sensitivities. Finally, we integrated the *N18* mutation at the genomic locus in a wild-type strain using the method illustrated in S5 Fig B. As a control, we integrated wild-type *tti1* using the same method. We found that while the wild-type *tti1* integrant was resistant to HU and MMS, two separate colonies from the *N18* integration were sensitive to HU and MMS (Fig 2C, top panels). Compared to the primary *N18* mutant, two individual colonies appeared to be slightly more sensitive. This is likely due to the epitope tagging, which may slightly affect the *tti1* function. We also examined the sensitivities of *N18* to other DNA-damaging agents, camptothecin, UV, and bleomycin (Fig 2C, bottom panel). The results showed that *N18* and the *N18* integrants were also sensitive to these agents. Together, we conclude that the *tti1* mutations in *N18* and the six other mutants affect the Rad3 kinase signaling function in the DRC and DDC, causing genotoxin sensitivities. Hereafter, we refer to *N18* as the *tti1-N18* mutant in the rest of the study.

### The *cut* phenotype in HU-treated *tti1* mutants

**Fig 3:**
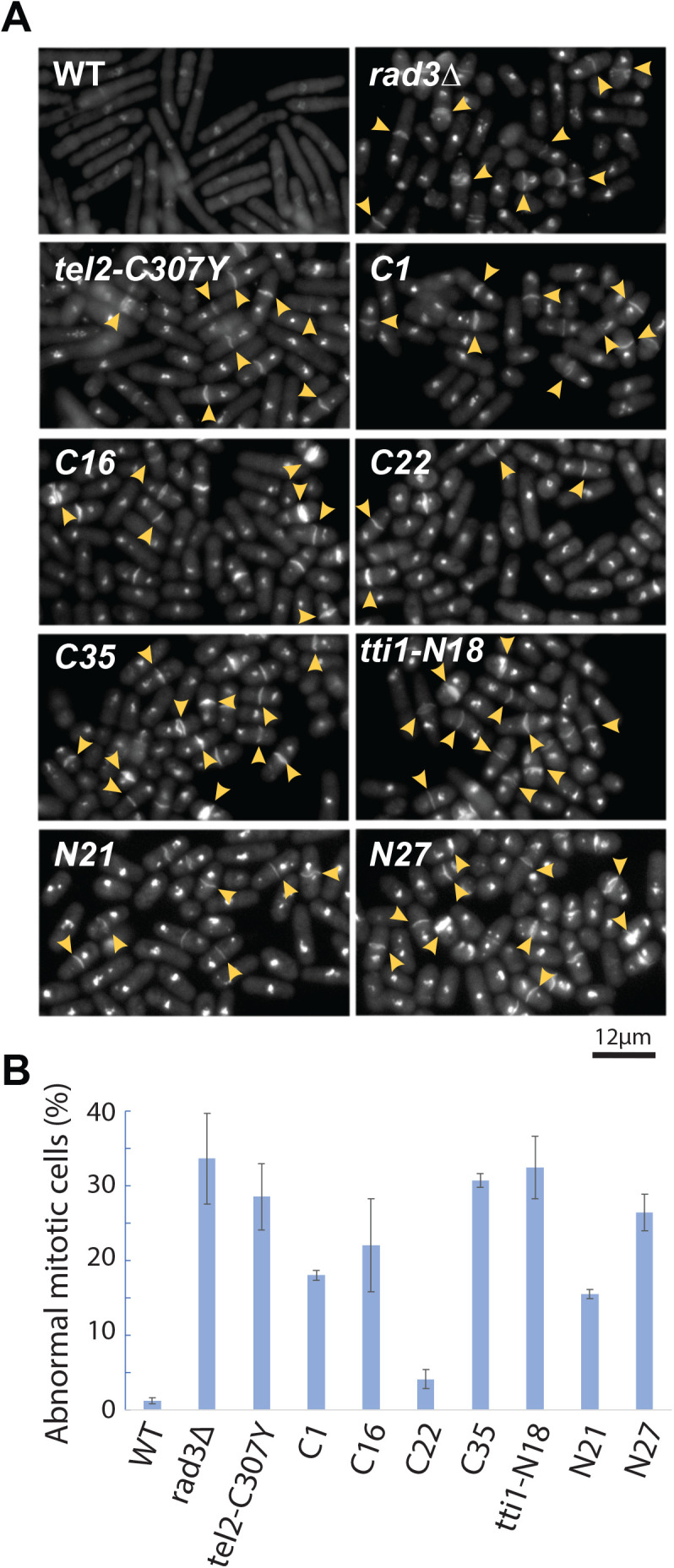
Microscopic examination of the *cut* cells in HU-treated *tti1* mutants. **(A)** Wild-type *S. pombe*, *rad3∆, tel2-C307Y,* and the indicated *tti1* mutants were treated with 15 mM HU for 6 h, fixed onto glass slides, and then stained with Hoechst and Blankophor for detecting DNA and septum, respectively. The stained cells were examined under the microscope. The arrows indicate the *cut* cells. **(B)** About 250 cells were counted for each sample, repeated three times, and the data are presented in percentages.

The *cut* (cell untimely torn) refers to premature mitosis in which the genetic material is unequally distributed into the dividing cells, a phenotype typically observed in checkpoint mutants treated with HU [41,42]. To further investigate the checkpoint defect, we examined the HU-treated *tti1* mutants under a microscope after staining the DNA with Hoechst and the septum with Blankophor (Fig 3A). When treated with 15 mM HU for 6 h, wild-type cells were mononuclear and elongated, consistent with the functional checkpoints. Since the *rad3∆* cells lack the DRC and DDC, the cells were short, and a large number of cells exhibited the *cut* phenotype (arrows). Although the *tel2-C307Y* cell lacks the DRC, it has a partially functional DDC. Therefore, *cut* and slightly elongated cells were observed. Under similar conditions, all *tti1* mutants, except *C22*, showed a significant increase in *cut* cells (Fig 3B). Since the HU-treated *C22* cells are shorter than wild-type cells and behave like the metabolic mutants we have reported previously [43,44], *C22* may be under the oxidative stress induced by HU, which arrests the cells at G2. Together, these results showed that all newly screened *tti1* mutants, except C22, are significantly defective in the checkpoint pathways.

### The *tti1-N18* mutation eliminates the function of Rad3^ATR^, but not Tel1^ATM^

**Fig 4:**
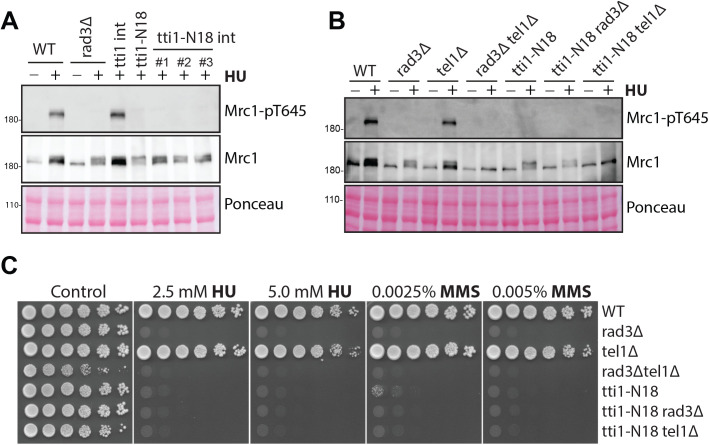
The *tti1-N18* mutation abolishes the kinase signaling function of Rad3^ATR^, but not that of Tel1^ATM^. **(A)** Rad3 phosphorylation of Mrc1-T645 in three separate colonies of the *tti1-N18* integration was examined by Western blotting, as in Fig 1D, except the gel was run for a longer time. Wild-type cells, the mutants of *rad3∆, tti1-N18*, and the integrants of wild-type *tti1* and *tti1-N18* were used as the controls. **(B)** Phosphorylation of Mrc1 by Rad3 and Tel1 was examined by using a phospho-specific antibody and mobility shift assay, respectively. Wild-type cells, mutants of *rad3∆, tel1∆, tti1-N18*, and the double mutants of *rad3∆ tel1∆*, *tti1-N18 rad3∆,* and *tti1-N18 tel1∆* were treated with 15 mM HU for 3 **h.** The Rad3-specific phosphorylation of Mrc1-T645 (upper panel) and Tel1-dependent mobility shift of Mrc1 in *rad3∆* cells (middle panel) were detected in whole cell lysates by Western blotting as in **A. (C)** HU sensitivity of wild-type *S. pombe*, *rad3∆*, *tel1∆*, *tti1-N18* mutants, and the double mutants of *tti1-N18 rad3∆* and *tti1-N18 tel1∆* was examined by spot assay.

Using the *tti1-N18* integrants, we re-examined the mutational effect of *tti1-N18* on Rad3 and Tel1 by running the gel for a longer time to achieve better separation (Fig 4A). Similar to Fig 1D, wild-type cells and the integrant of wild-type *tti1* showed similarly increased levels of Mrc1 protein and Mrc1-T645 phosphorylation after HU treatment. In *rad3∆* cells, phosphorylation of Mrc1-T645 is eliminated as expected. However, the Tel1-dependent phosphorylation, as evidenced by the mobility shift of Mrc1, remained in *rad3∆* cells. In the *tti1-N18* mutant and the integrant, the Rad3-dependent Mrc1-T645 phosphorylation was eliminated, whereas the Mrc1 mobility shift was detected as in *rad3∆* cells. This suggests that Tel1 remains functional in *tti1-N18*.

Mrc1 is phosphorylated by both Rad3 and Tel1 in HU [33]. Consistent with the phosphorylation, Mrc1 possesses 14 S/TQ sites, and among which, T645 and T653 are phosphorylated specifically by Rad3 [33,34]. Since Tel1-specific phosphorylation sites in Mrc1 have not yet been identified, we used the Tel1-dependent mobility shift to assess Tel1 kinase activity in HU described above. To further investigate, we crossed *tti1-N18* into *rad3∆* or *tel1∆* cells and examined the Mrc1-T645 phosphorylation and Mrc1 mobility shift (Fig 4B). Unlike *rad3∆* cells, phosphorylation of Mrc1-T645 and the mobility shift remained in *tel1∆* cells, confirming the Rad3-dependent phosphorylation. In the double mutant of *rad3∆tel1∆,* both the Mrc1-T645 phosphorylation and the mobility shift were eliminated. Similar to Fig 4A, while the phosphorylation of Mrc1-T645 was eliminated in *tti1-N18* or the double mutant of *tti1-N18 rad3∆,* the mobility shift remained in *tti1-N18* and the double mutant of *tti1-N18 rad3∆*. In the double mutant of *tti1-N18 tel1∆* cells, neither Mrc1-T645 phosphorylation nor the mobility shift was detected, as in *rad3∆tel1∆* cells. Together, these results demonstrate that the *tti1-N18* mutation specifically affects the kinase activity of Rad3^ATR^, not Tel1^ATM^, in the presence of HU. We then assessed the drug sensitivities of *tti1-N18* and the double mutants of *tti1-N18 tel1∆* and *tti1-N18 rad3∆* (Fig 4C), and the results are consistent with the phosphorylation defects observed in Fig 4A and B.

### Moderately reduced or unchanged levels of PIKK proteins in the *tti1-N18* mutant

**Fig 5:**
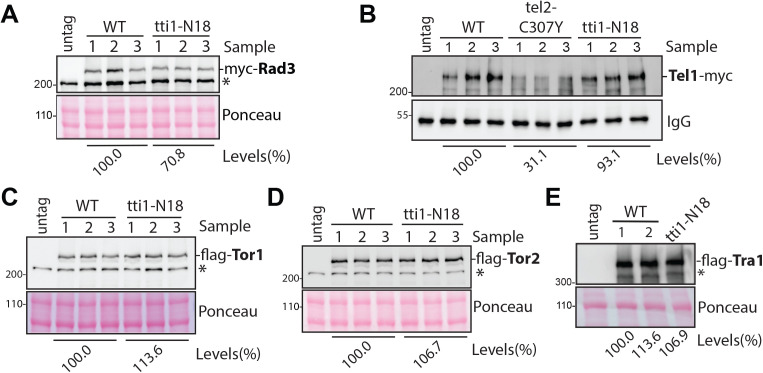
The protein levels of PIKKs were moderately reduced or unaltered in *tti1-N18.* **(A)** Rad3 was detected in whole cell lysates using anti-myc antibody (top panel). Asterisk indicates a cross-reacting material. A section of Ponceau S-stained membrane was shown as the loading control (lower panel). Three samples were individually prepared for each indicated strain. The Rad3 bands were quantified and shown at the bottom. **(B)** Tel1 was IPed from wild-type, *tel2-C307Y,* and *tti1-N18* cells for measuring the protein levels by Western blotting. The section of blot with IgG used for the IP is shown in the lower panel. (**C)** Tor1 was flag-tagged in wild-type and the *tti1-N18* mutant cells at the genomic locus. Whole-cell lysates were separated by SDS-PAGE followed by Western blotting using anti-flag antibody. The asterisks indicate a cross-reactive material. A section of Ponceau S-stained membranes is shown as loading controls (lower panels). **(D**) The protein levels of the flag-tagged Tor2 were examined as in **C. (E)** Tra1 was detected in whole-cell lysates by Western blotting after being separated in a 5% SDS-PAGE gel. The asterisk indicates a cross-reactive material. A section of the Ponceau S-stained membrane is shown.

Since the TTT regulates PIKK maturation, we examined the steady-state protein levels of Rad3, Tel1, and other PIKKs. Similar to the moderately decreased Rad3 level in *tel2-C307Y* [28], a moderate reduction in Rad3 protein level was observed in the whole cell lysates of *tti1-N18* (Fig 5A). Since the Tel1 level is low, we IPed Tel1 for the examination, and found that while Tel1 was significantly reduced in *tel2-C307Y* mutant [28], it was only slightly reduced in *tti1-N18* (Fig 5B), which is consistent with the functional Tel1 shown in Fig 4.

As mentioned above, fission yeast possesses four other PIKKs: Tor1 and Tor2, the homologs of mammalian mTOR, and Tra1 and Tra2, the homologs of TRRAP, but lacks the homologs of mammalian DNA-PKcs and SMG1. Tor1 acts via TORC2 to modulate stress responses, differentiation, and lifespan under nutrient-limiting conditions, while Tor2, a component of TORC1, is essential for cell growth and metabolism [45]. Tra1 and Tra2 are involved in transcriptional regulation and genome stability [46,47]. To investigate whether the *tti1-N18* mutation affects other PIKKs, we measured the levels of Tor1, Tor2, and Tra1. Tra2 level was not examined because an epitope-tagged *tra2* strain is not available, and, as shown below, its function is likely unaffected in *tti1-N18*. As shown in Fig 5C, D, and E, the protein levels of both Tor1, Tor2, and Tra1 were unreduced in *tti1-N18*.

### Uncompromised functions of other PIKKs in *tti1-N18*

**Fig 6:**
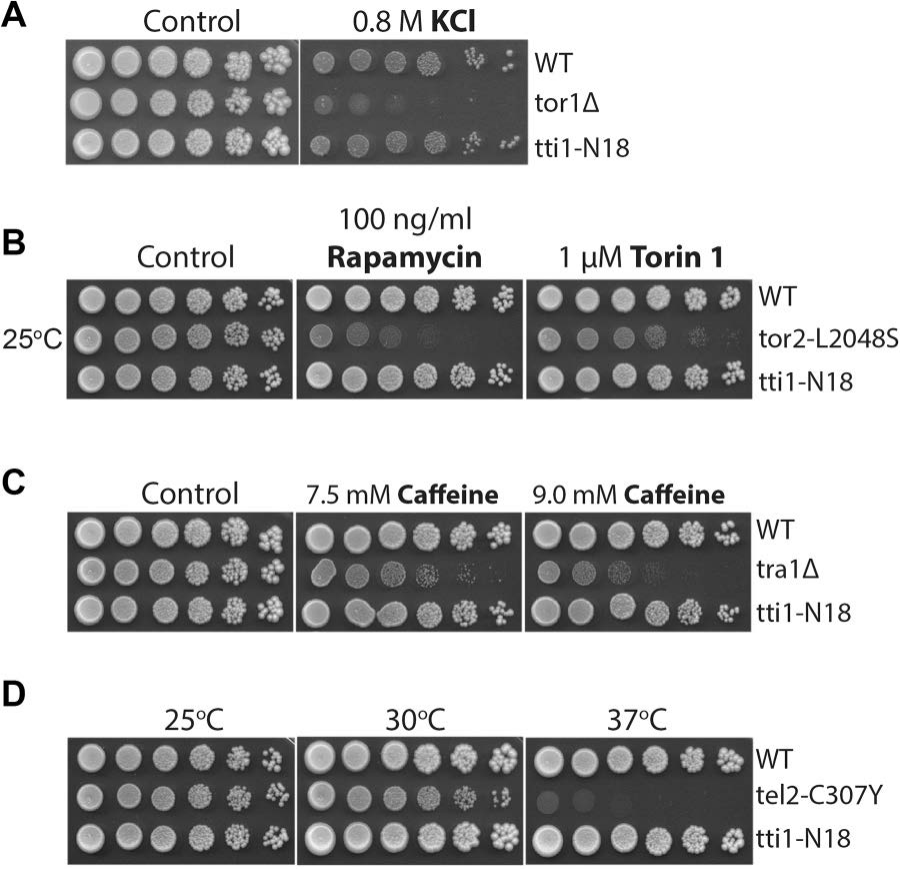
Uncompromised functions of other PIKKs in *tti1-N18.* **(A)** The sensitivities of wild-type, *tor1∆,* and *tti1-N18* cells to 0.8 M KCl were examined by spot assay. While the control plate was incubated at 30˚C for 3 days, the plate containing KCl was incubated at 30˚C for 6 days before photographing. **(B)** The sensitivities of wild-type, *tor2-L2048S,* and *tti1-N18* cells to Rapamycin and Torin at the indicated concentrations were examined. All plates were incubated at the permissive temperature of 25˚C for 4 days. **(C)** Sensitivities of wild-type, *tra1∆,* and *tti1-N18* cells to caffeine at the indicated concentrations were examined by spot assay. **(D)** The cell growth of wild-type *S. pombe* and the mutants of *tel2-C307Y* and *tti1-N18* was examined by spot assay at the indicated incubation temperatures.

The unchanged protein levels of Tor1, Tor2, and Tra1 prompted us to investigate whether the other PIKKs are functional, because the proteins, even though expressed at wild-type levels, may not be functional due to improper folding. Spot assay showed that the cells lacking Tor1 were sensitive to 0.8 M KCl [48,49] (Fig 6A). Under similar conditions, the *tti1-N18* mutant behaved like the wild-type cells, suggesting that Tor1 remains functional in the mutant. Since Tor2 is essential for cell growth, we examined the temperature-sensitive (*ts*) mutant *tor2-L2048S*. We found that at the permissive temperature 25˚C, the *tor2-L2048S* mutant was sensitive to Rapamycin and Torin [50,51] (Fig 6B). Under similar conditions, *tti1-N18* was resistant like the wild-type cells. We then investigated whether the cellular functions of Tra1 and Tra2 were affected. The spot assay showed that *tra1∆* cells were sensitive to caffeine [52](Fig 6C). However, the *tti1-N18* cells showed resistance like wild-type cells under similar conditions. Since the *tti1-N18* colonies were slightly smaller than wild-type cells in caffeine, we then examined the sensitivity to higher doses of caffeine (S6 Fig). The results showed that while the cell growth of the *tti1-N18* as well as the FLAG-tagged *tor1* and *tra1* strains was slightly suppressed by 10 or 15 mM caffeine, the *tra1∆* cells were more sensitive to caffeine. Among the six PIKKs in fission yeast, only Tor2 and Tra2 are essential for cell survival, which likely explains the *ts* phenotype of the *tel2-C307Y* mutant [28]. Since the function of Tor2 is uncompromised in *tti1-N18* (Fig 6B), we examined whether *tti1-N18* is a *ts* mutant, assuming that if it is, the *ts* phenotype must be due to functional loss of Tra2. As shown in Fig 6D, unlike *tel2-C307Y*, the *N18* mutant grew like wild-type cells at all temperatures tested, confirming that it is not a *ts* mutant. Since a non-lethal *tra2* mutant is not available in fission yeast, which prevents a functionality assay, these results, nonetheless, show that, except Rad3, the functions of all other PIKKs, including Tel1, were largely unaffected in *tti1-N18*.

We also examined *tti1-C35* and *tti1-N27* and found that both mutants, particularly *C35*, grew more slowly at 25˚C and 37 ˚C, suggesting the function of either Tor2 or Tra2 might be affected (S7, Fig A). The TTT complex revealed by cryo-EM shows that the three subunits Tti1, Tel2, and Tti2 form the complex at a 1:1:1 stoichiometry [15,16]. Previous study of the *tel2-C307Y* mutant demonstrated a partially destabilized TTT with severe impairment of Tel2 interactions with Tti1 and Tti2 [28]. AlphaFold3 modelling suggests that the *C35* mutation altered the TTT structure significantly [S7 Fig B, compare wild-type TTT (top) with TTT with C35 mutation (lower)]. To further investigate, we examined the sensitivity of *C35* and *N27* to a high dose of KCl (S7 Fig C), Rapamycin (S7 Fig D), and Caffeine (S7 Fig E). The results showed that while *tel2-C307Y* was highly sensitive to Rapamycin and Caffeine, indicating defective Tor2 and Tra1, it showed a wild-type resistance to KCl, suggesting a functional Tor1. The *C35* was found to be resistant to KCl but sensitive to Rapamycin and Caffeine, suggesting that Tor2 and Tra1, not Tor1, were affected in *C35,* consistent with a broader effect of *C35* mutation on PIKKs. In contrast, *N27*, like *tti1-N18*, showed wild-type resistance to all three agents tested, suggesting that the functions of Tor1, Tor2, Tra1, and Tra2 were not or minimally affected in *N27*.

### The *tti1-N18* mutation moderately destabilizes the TTT complex

**Fig 7:**
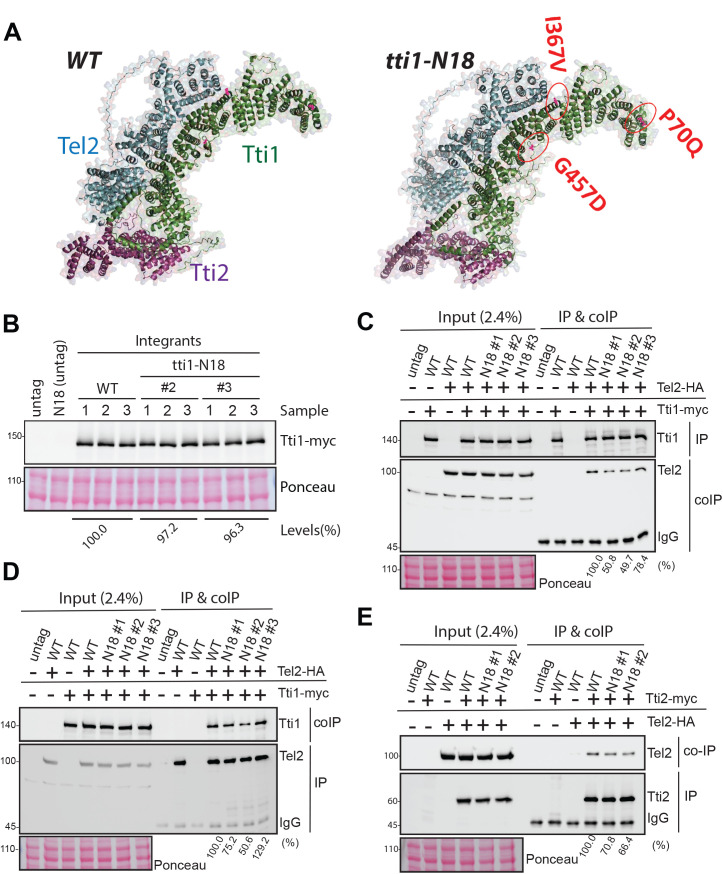
The TTT complex is moderately destabilized in *tti1-N18.* **(A)** Minimal perturbation of the overall structure of the TTT by *tti1-N18* mutation. AlphaFold modelling of the complex containing wild-type Tti1 (left) or Tti1-N18 (right), respectively. The three subunits of the TTT are coloured in green for Tti1, blue for Tel2, and purple for Tti2. The three mutated residues, P70, I367, and G457, are coloured in red in wild-type TTT and circled in the mutant TTT. **(B)** Wild-type *tti1* and the mutant *tti1-N18* were tagged with myc epitope at the genomic locus. The whole cell lysates were analysed by SDS-PAGE and Western blotting using anti-myc antibody. To ensure accuracy, three separate samples were prepared for each indicated strain. A section of the Ponceau S-stained membrane is shown as a loading control (bottom panel). Quantitation results are shown at the bottom. **(C)** Co-IP of Tel2 with Tti1 was moderately reduced in *tti1-N18*. Tti1 was IPed (upper panel) using anti-myc antibody to detect co-IPed Tel2 (lower panel). Three separate samples of *tti1-N18* were used to ensure accurate quantification. 2.4% of the whole cell extracts were analysed as inputs (left seven lanes). A section of the Ponceau S-stained membrane for the inputs is shown. **(D)** Tel2 was IPed from the same strains used in **C** with an anti-HA antibody to detect the co-IPed Tti1 in three individual samples of *tti1-N18* mutant. **(E)** Tti2 was IPed using anti-myc antibody as in **C.** The co-IPed Tel2-HA was detected by Western blotting in wild-type and two separate samples of the *tti1-N18* mutant.

Since Tel2 interacts with the central region of Tti1, where two of the three *tti1-N18* mutations are located, we investigated whether the mutations affect the stability of the TTT. AlphaFold3 modelling showed that the three-point mutations in *tti1-N18* mildly altered the overall structure, as compared with the TTT with C35 mutation (compare Fig. 7A with S7 Fig B). To validate the modelling result, we performed the co-immunoprecipitation (co-IP) assays to assess the stability of the TTT in *tti1-N18*.

We tagged *Tti1* with a myc epitope at the C-terminus in the genomic locus (S5 Fig B). Western blotting showed that under physiological conditions, the Tti1-N18 mutant was expressed at a wild-type level (Fig 7B), suggesting that the elimination of Rad3 activity is likely due to a functional defect of the mutant Tti1. We then IPed Tti1 using anti-myc antibody agarose beads to examine whether the mutation affects the coIP of Tel2. The results showed a partial reduction in the coIPed Tel2 in *tti1-N18* (Fig 7C). In the reciprocal coIP where Tel2 was IPed using anti-HA antibody beads (Fig 7D), a similar reduction of the coIPed Tti1 was found, confirming a partial defect in the Tti1-Tel2. To investigate the interaction of Tel2 and Tti2, Tti2 was IPed with anti-myc antibody to monitor the coIPed Tel2. We found that although the mutated residues in Tti1 do not directly interact with Tti2, the mutation also moderately reduced the level of coIPed Tel2 with Tti2 (Fig 7E). Together, we show that the *tti1-N18* mutation partially compromises the interactions among the three subunits of the TTT. This result is consistent with the non-lethality of *tti1-N18* and the mechanism by which the three subunits of the TTT act as a single unit. In addition, tetrad dissection showed that both the primary mutant and the *tti1-N18* integrant are synthetic lethal with the *tel2-C307Y* mutations (S8 Fig).

## Discussion

Rad3^ATR^ is highly conserved in eukaryotes. It is one of the six PIKKs and the master regulator of the DRC and DDC pathways in fission yeast [26]. The diverse biological functions of the PIKKs, including Rad3, are all controlled by the conserved TTT complex comprising Tel2, Tti1, and Tti2. The current model suggests that the TTT plays a crucial role in the maturation and stabilization of newly synthesized PIKKs [11,12,17]. It remains unclear, however, whether the TTT or its subunits have other cellular functions, as the disease phenotypes of the TTT are different from those of the PIKK mutations. Our previous characterization of the *tel2-C307Y* mutation in fission yeast showed that, in addition to PIKK maturation, the TTT may regulate the DRC pathway downstream of Rad3 [28]. To investigate this possibility, we employed a targeted forward genetic approach, focusing on the largest subunit of the TTT, and screened a series of *tti1* mutants that are sensitive to genotoxins. The initial screenings identified nearly 100 primary mutants. Further characterization of these primary mutants by genetic crosses, complementation analysis, extrachromosomal expression, and DNA sequencing removed a large number of redundant and non*-tti1* mutants, such as the metabolic mutations that sensitize the cells to HU [43,44]. Ultimately, we identified seven *tti1* mutants *C1*, *C16*, *C22*, *C35*, *N18*, *N21*, and *N27* (Table 1 and Fig 1).

Characterization of the seven *tti1* mutants revealed varying degrees of impaired DRC and DDC pathways, as evidenced by reduced or eliminated Rad3^ATR^-dependent phosphorylation of Mrc1, Cds1, and Chk1. In general, these Rad3^ATR^ signaling defects corroborate drug sensitivities (Fig 1A and B) and the number of *cut* cells observed in these mutants (Fig 3). The small number of *cut* cells and short cell length observed in *C22* are likely due to the oxidative stress induced by HU, like the metabolic mutants we have previously reported [43,44]. Therefore, the identified *tti1* mutations compromise or eliminate Rad3 kinase signaling, leading to the observed genotoxic sensitivities. According to the current model, the *tti1* mutations likely affect the function of the TTT and thus the misfolding of the PIKKs. Consistent with this model, we found that the *tel2-C307Y* is a *ts* mutation that nearly eliminated the Rad3^ATR^ phosphorylation and partially compromised Tel1^ATM^ phosphorylation of Mrc1 in HU. It also significantly compromised the functions of Tor2 and Tra1 (S7 Fig D and E). Remarkably, the *C35* mutation eliminated the kinase functions of both Rad3^ATR^ and Tel1^ATM^ (Fig 1D) and mildly affected the functions of Tor2 and Tra1 (S7 Fig D and E).

Unexpectedly, unlike the *tel2-C307Y* and the *C35* mutants that have a broad effect on the PIKKs, the *tti1-N18* mutation, as well as *N27* that shares two mutated residues with *tti1-N18*, specifically eliminated the function of Rad3^ATR^, but not that of Tel1^ATM^ (Fig 4). Furthermore, the functions of four other PIKKs are largely intact in the mutant. The co-IP experiments and AlphaFold modeling showed that, similar to the *tel2-C307Y* mutant, the *tti1-N18* mutation partially destabilizes the TTT, suggesting that the Rad3^ATR^ function was specifically eliminated through the destabilized TTT. It remains unclear, however, why the partially destabilized TTT compromises the function of Rad3, Tel1, Tor2, and Tra1 in *tel2-C307Y* [28]*,* while the partially destabilized TTT in *tti1-N18* mainly eliminates the function of Rad3, not that of other PIKKs. One possibility is that, unlike the current model [44], the TTT recognizes the premature PIKKs with substrate specificity. Mutations that affect the specificity allow the selective elimination of the function of a particular PIKK, not all PIKKs. Consistent with this possibility, the three mutated residues in *tti1-N18* are all located on the surface of the TTT (Fig 7A), where premature Rad3 may bind for proper folding. This notion is supported by *C35,* whose mutation alters the TTT structure more significantly and affects the specificity more broadly than the *tti1-N18* mutation. The non-lethality of the *C35* mutant also suggests that the mutation does not eliminate the function of the TTT because the functions of Tor2 and Tra2 are required for cell survival. Alternatively, different PIKKs may vary in their expression levels and turnover rates, which determine their cellular functions. Rad3 may be expressed at higher levels or has a faster turnover rate than other PIKKs, leading to its observed loss-of-function in *tti1-N18* with partially dysfunctional TTT. Further studies on Tra1, Tra2, Tor1, and Tor2 in *tti1-N18*, such as the transcription-based analyses, should reveal more molecular details of the regulatory functions of TTT on PIKKs. Nevertheless, although this work has not addressed the question of whether the TTT regulates the DRC downstream of Rad3, it provides further insights into the role of the TTT in coordinating Rad3-mediated checkpoint responses in fission yeast.

Although the mechanism by which the TTT acts as a co-chaperone of Hsp90 for PIKK maturation appears to be well established, some molecular details remain incompletely understood. For example, the TTT requires additional cofactors for stabilization and the proper folding of PIKKs. In budding yeast and mammalian cells, the super complex of TTT-Hsp90-R2TP facilitates the maturation of PIKKs with additional proteins Tah1 and Pih1 that bridge TTT with R2TP [53,54]. However, homologs of Tah1 and Pih1 have not been identified in *S. pombe*. Identification of their functional homologs in fission yeast would provide more insights into the mechanism of the TTT-mediated PIKK maturation. Nonetheless, the essential function of the TTT in PIKK maturation makes it an important target for cancer chemotherapy. In support of this notion, human Tel2 has been identified as a target of Ivermectin, an FDA-approved antiparasitic drug [24,25], which raises the possibility of repurposing this clinically established drug for cancer treatment. Given that the TTT may facilitate the maturation of PIKKs in a substrate-specific manner, as demonstrated here in the *tti1-N18* mutant, targeting the TTT for eliminating a particular set of PIKK pathways may represent a promising therapeutic strategy.

## Conclusion

Our targeted forward genetic screen has discovered seven *tti1* mutants that are sensitive to genotoxins, highlighting the central role of Tti1 in the co-translational maturation of PIKKs. A specific *tti1* mutant has been identified that selectively eliminates the checkpoint function of Rad3^ATR^ while leaving other PIKKs, including Tel1^ATM^, largely unaffected. These results not only advance our understanding of the essential function of the TTT complex but also open new therapeutic opportunities for the treatment of cancer or other diseases.

## Materials and methods

### Yeast strains and plasmids

The *S. pombe* strains used in the study were cultured in YE6S liquid media containing 0.5% yeast extract, 3% dextrose, with six supplements (adenine, uracil, arginine, lysine, leucine, and histidine) or in EMM6S media. The yeast strains, plasmids, and PCR primers used in the study are listed in S1–S3 Tables. The mutations were identified by DNA sequencing (Retrogen).

### Genetic screen of the *tti1* mutants

The targeted forward genetic screen using the pop-in and pop-out method was carried out following the previously described method [29,30] as illustrated in S1 Fig.

### Western blotting

The protein levels of Rad3, Tel1, Tor1, Tor2, and Tra1 were examined by tagging these proteins with myc, HA, or FLAG epitope at the genomic locus and Western blotting using antibodies against myc (ThermoScientific), HA, or FLAG (Sigma). Rad3-dependent phosphorylation of Mrc1 and Cds1 was detected using phospho-specific antibodies as described previously [34]. The Rad3-dependent phosphorylation of Chk1 was determined by mobility shift assay [37]. The blotting signals were detected by enhanced chemiluminescence and imaged using ChemiDoc XRS imaging system (Bio-Rad).

### Microscopy

The cells were fixed onto glass slides by briefly heating at 75˚C. The fixed cells were stained in PBS buffer containing 5 µg/ml Hoechst33258 (Sigma-Aldrich) and diluted Blankophor stock solution (MP Biochemicals). The stained cells were examined using an Olympus EX41 fluorescent microscope. Images were captured with an IQCAM camera (Fast1394) using Qcapture Pro 6.0 software and then extracted into Photoshop (Adobe) to generate the figures.

### AlphaFold modeling

The protein sequences of *S. pombe* Tel2, Tti1, and Tti2 were submitted to the Alphafold3 server (https://alphafoldserver.com/). The resulting models of the TTT complex structures with wild-type Tti1, Tti1-N18, and Tti1-C35 mutant proteins were analyzed in PyMOL (The PyMOL Molecular Graphics System, Version 3.0, Schrödinger, LLC).

### IP and co-IP

1 x 10^8^ logarithmically growing cells were harvested in 1.5 ml screw cap tubes and saved at -20 ˚C as previously described [28]. The cell pellets were lysed with a mini-bead beater using the lysis buffer of 25 mM HEPES/NaOH at pH 7.5, 1 mM NaVO_4_, 10 mM Na_4_P_2_O_7_, 50 mM NaF, 40 mM ß-glycerophosphate, 0.1% Tween 20, 0.5% NP-40, and protease inhibitors. The cell lysates were centrifuged at 16,000 g, 4 ˚C for 5 min. The clarified extracts were incubated with prewashed antibody agarose resins or magnetic Dynabeads at 4 ˚C for 2 h. After washing three times, the agarose resins or Dynabeads samples were separated by 6% or 8% SDS-PAGE, followed by Western blotting.

## Supporting information

S1 Fig Genetic screening of new *tti1* mutants via genomic allele replacement. (A) Construction of the *tti1* expression cassette. The *tti1* N- and C- terminal regions, along with engineered restriction sites NdeI, NheI, and XmaI via silent mutations, are labelled. Random mutations by error-prone PCRs were generated between NdeI and NheI, and NheI and XmaI, creating two separate *tti1* N- and C- terminal mutant libraries. (B) Strategy for integrating the mutations at the *tti1* genomic locus. After plasmid linearization through enzymatic digestion, the library DNAs were transformed into a wild-type *S. pombe* strain lacking the *ura4* gene. The *ura4* pop-in transformants were sequentially cultured in EMM6S liquid media lacking uracil to eliminate non-transformed cells and the cells with lethal mutations. To pop-out the *ura4* marker in the second step, the *ura4* positive transformants were cultured in YE6S rich media until saturation to allow for the pop-out of *ura4*, followed by counter selection on 5-FOA plates. The *ura4* negative colonies formed on 5-FOA plates carry either the wild-type or mutant *tti1* at the genomic locus. The colonies were then screened for sensitivity by replica plating on HU and MMS plates. The drug-sensitive mutants were streaked out into single colonies, confirming drug sensitivities. (C). Representative *tti1* mutants screened by using the N- and C- terminal libraries were assessed by three-spot assays. The drug-sensitive mutants were backcrossed, confirmed by sequencing, and subsequently renamed for the experiments described in this study.
(TIF)

S2 Fig Identification of the mutations of the seven *tti1* mutants by Sanger sequencing. The mutations were identified by sequencing for each mutant. The mutants, shown in A-G, are listed on the left side. All mutation sites are highlighted in blue. Nucleotide changes are marked above the blue highlights. Corresponding amino acid substitutions are denoted in bold. The flanking short, unchanged amino acid sequences are also shown.
(TIF)

S3 Fig Distribution of the mutated residues along the Tti1 molecule. The Tti1 amino acid sequences from fission yeast (*Sp*), budding yeast (*Sc*), and humans (*Hs)* were aligned together using MacVector. The mutated residues in *S. pombe* Tti1 are marked by dots. While the blue dots denote highly conserved residues, green dots and pink dots indicate partial and minimal conservation, respectively. The red asterisk marks a stop codon to present a truncation mutation in the *C22* mutant. The three mutations in the *tti1-N18* mutant are highlighted in green squares.
(TIF)

S4 Fig Tetrad dissection confirms the *tti1* mutations in six mutants. Wild-type *S. pombe* or a wild-type strain in which *tti1* is linked to the *kanR* marker was crossed with *tti1-C1, C35* (A), *C22, C16* (B), and *N21, N27* (C) mutants. Tetrad dissection was performed for each cross, and the colonies formed on YE6S plates were replica plated on HU and MMS plates containing the lethality dye phloxine B to reveal the mutations, YE6S plates containing G418 to show the *kanR* marker, and low adenine plates to reveal the two alleles of ade6 as indicated by red or pink colors. All tetrads from each cross showed a 2:2 ratio of *kanR*, ade6 alleles, and drug sensitive phenotype. The drug phenotype is always segregated from the *kanR* marker in all crosses, which confirms the successful dissection and the *tti1* mutations in all six mutants examined. Tetrad dissection for *N18* is shown in Fig 2A.
(TIF)

S5 Fig Rescuing effect of wild-type Tti1 in all seven *tti1* mutants and the strategy for integrating *tti1-N18* at the genomic locus. (A) The *tti1* mutants were transformed with an empty vector (v) or a vector expressing the wild-type Tti1. Cells were sequentially diluted in ten-fold steps and spotted on YE6S or plates containing HU or MMS at the indicated concentrations. Wild-type *S. pombe*, *rad3∆, cds1∆,* and *chk1∆* mutants were used as controls. Expression of Tti1 rescued the drug sensitivity and supports the conclusion that all seven *tti1* mutants were caused by mutations in *tti1*. (B) 9myc and nmtT represent the myc epitope tag and *nmt1* terminator, respectively. Integrants were screened by colony PCR to ensure successful integration into the genome. Genomic DNA was purified for PCR to confirm integration at the *tti1* locus by Sanger sequencing. Western blotting using anti-myc antibody to confirm a protein band of the expected size for Tti1.
(TIF)

S6 Fig Higher doses of Caffeine slightly suppresses the cell growth of *tti1-N18.* Sensitivities of wild-type *S. pombe*, tagged strains, and the mutants with the indicated mutations to Caffeine were examined by the three-spot assay. The plates were incubated at 30˚C for three days before being photographed. Two separate colonies of *tra1∆* were examined. The dashed line indicates discontinuity.
(TIF)

S7 Fig The *ts* phenotype, AlphaFold3 modeling, and the sensitivities of *C35* and *N27* to KCl, Rapamycin, and Caffeine. (A) Wild-type *S. pombe* and the mutants of the indicated mutations were spotted on YE6S and incubated at 25˚C, 30˚C, and 37˚C as in Fig 6D. Unlike the *tti1-N18* mutant that grew well under all tested temperatures, the *C35* and *N27* mutants showed a partial growth defect at 25˚C and 37˚C. (B) The structural impact of the *C35* mutation on the TTT complex revealed by AlphaFold3 modeling. The mutated residues were indicated in red in both the wild-type (top) and the mutated TTT complexes (bottom). Comparing with the TTT structure containing Tti1-N18 (Fig 7A), the *C35* mutation causes more significant changes to the TTT structure, which is consistent with the broader effect on PIKKs. Spot assay was used to examine the sensitivities of wild-type *S. pombe* and the indicated mutants to 0.8 M KCl (C), rapamycin (D), and caffeine (E).
(TIF)

S8 Fig The *tti1-N18* and *tel2-C307Y* mutations are synthetic lethal. The *tti1-N18:kanR* integrant and the *tti1-N18* primary mutant carrying the ade6-M210 allele were crossed with the *tel2-C307Y* mutant carrying the ade6-M216 allele. Tetrad dissection was performed on asci from the two crosses. Colonies formed on YE6S plates were replica-plated onto HU and MMS plates containing the lethality dye phloxin B, low adenine plates, and G418 plates to reveal the ade6 alleles and the kanR marker. The dissection results strongly suggest synthetic lethality or severe sickness.
(TIF)

S1 Table List of *S. pombe* strains used in this study. (PDF)

S2 Table List of plasmids used in this study. (PDF)

S3 Table List of PCR and sequencing primers used in this study. (PDF)
