# Supplementary figures and images for: A tti1 mutation in the Tel2-Tti1-Tti2 complex specifically eliminates the cellular function of Rad3ATR, but not that of other PIKKs in fission yeast
Source: PLoS Genet. 2026 Jun 11;22(6):e1012206. doi: 10.1371/journal.pgen.1012206 (PMC13274921; doi:10.1371/journal.pgen.1012206)

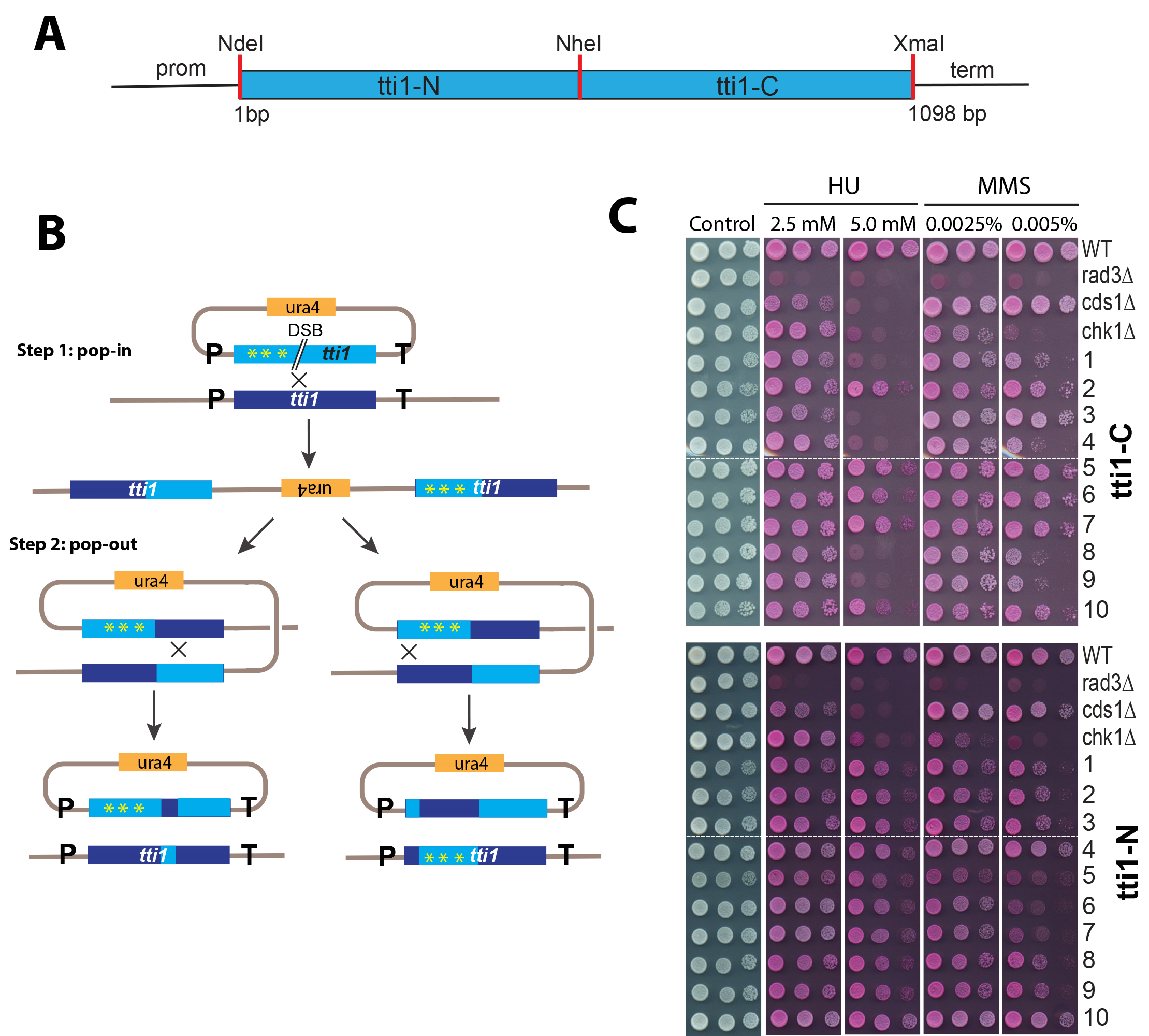

Supplement: S1 Fig — (A) Construction of the tti1 expression cassette. The tti1 N- and C- terminal regions, along with engineered restriction sites NdeI, NheI, and XmaI via silent mutations, are labelled. Random mutations by error-prone PCRs were generated between NdeI and NheI, and NheI and XmaI, creating two separate tti1 N- and C- terminal mutant libraries. (B) Strategy for integrating the mutations at the tti1 genomic locus. After plasmid linearization through enzymatic digestion, the library DNAs were transformed into a wild-type S. pombe strain lacking the ura4 gene. The ura4 pop-in transformants were sequentially cultured in EMM6S liquid media lacking uracil to eliminate non-transformed cells and the cells with lethal mutations. To pop-out the ura4 marker in the second step, the ura4 positive transformants were cultured in YE6S rich media until saturation to allow for the pop-out of ura4, followed by counter selection on 5-FOA plates. The ura4 negative colonies formed on 5-FOA plates carry either the wild-type or mutant tti1 at the genomic locus. The colonies were then screened for sensitivity by replica plating on HU and MMS plates. The drug-sensitive mutants were streaked out into single colonies, confirming drug sensitivities. (C). Representative tti1 mutants screened by using the N- and C- terminal libraries were assessed by three-spot assays. The drug-sensitive mutants were backcrossed, confirmed by sequencing, and subsequently renamed for the experiments described in this study. (TIF) [file pgen.1012206.s001.tif]

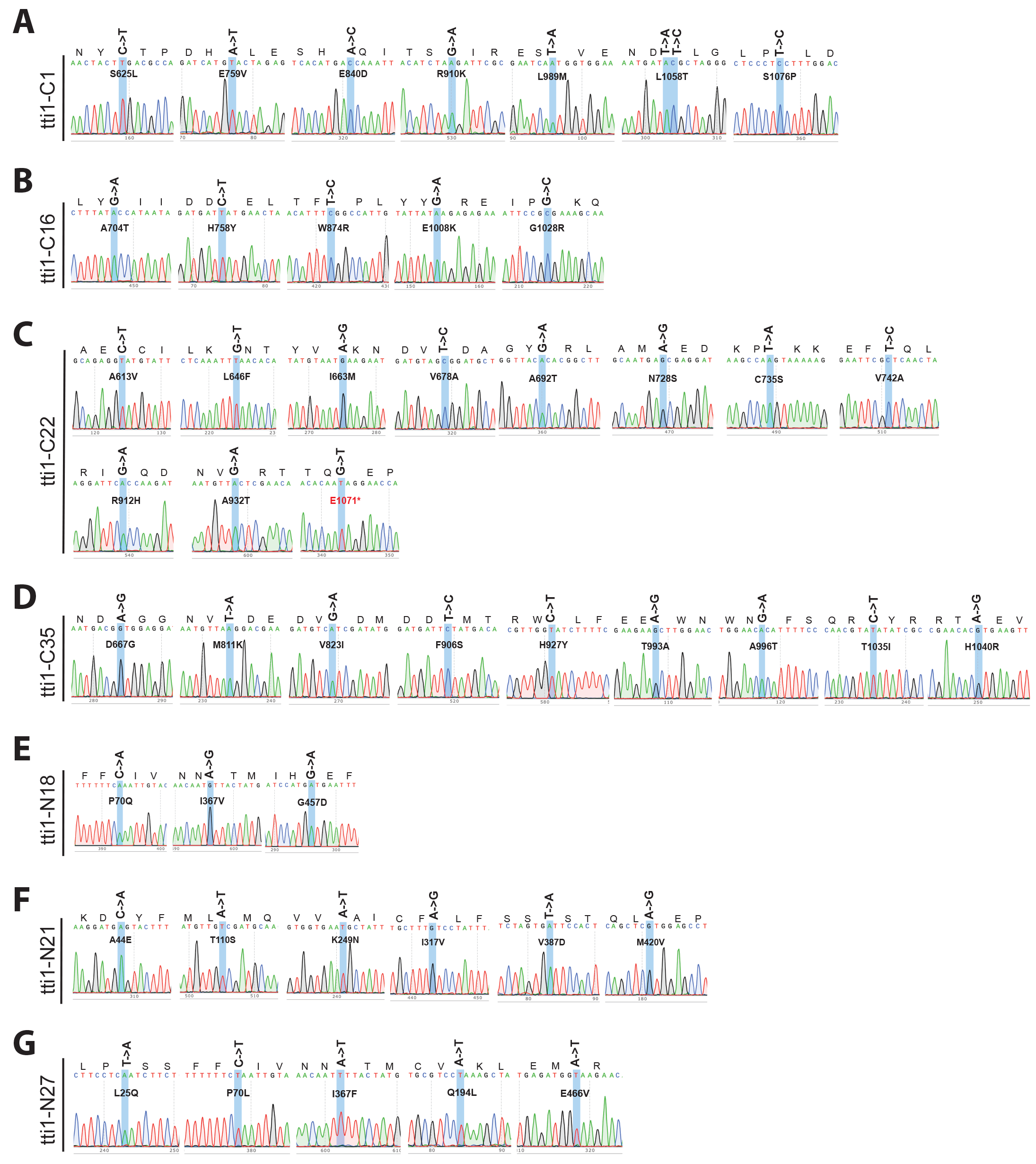

Supplement: S2 Fig — The mutations were identified by sequencing for each mutant. The mutants, shown in A-G, are listed on the left side. All mutation sites are highlighted in blue. Nucleotide changes are marked above the blue highlights. Corresponding amino acid substitutions are denoted in bold. The flanking short, unchanged amino acid sequences are also shown. (TIF) [file pgen.1012206.s002.tif]

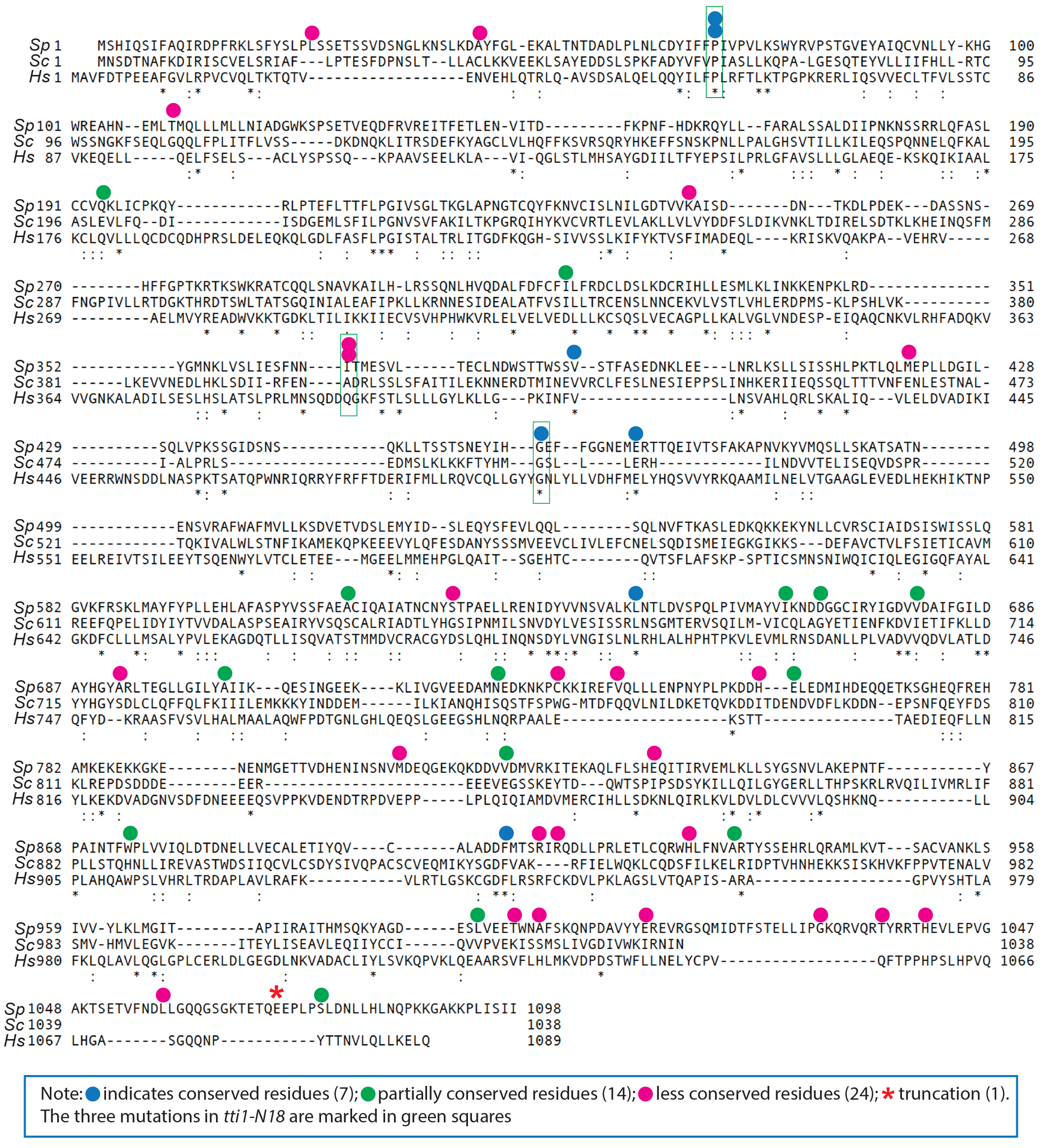

Supplement: S3 Fig — The Tti1 amino acid sequences from fission yeast (Sp), budding yeast (Sc), and humans (Hs) were aligned together using MacVector. The mutated residues in S. pombe Tti1 are marked by dots. While the blue dots denote highly conserved residues, green dots and pink dots indicate partial and minimal conservation, respectively. The red asterisk marks a stop codon to present a truncation mutation in the C22 mutant. The three mutations in the tti1-N18 mutant are highlighted in green squares. (TIF) [file pgen.1012206.s003.tif]

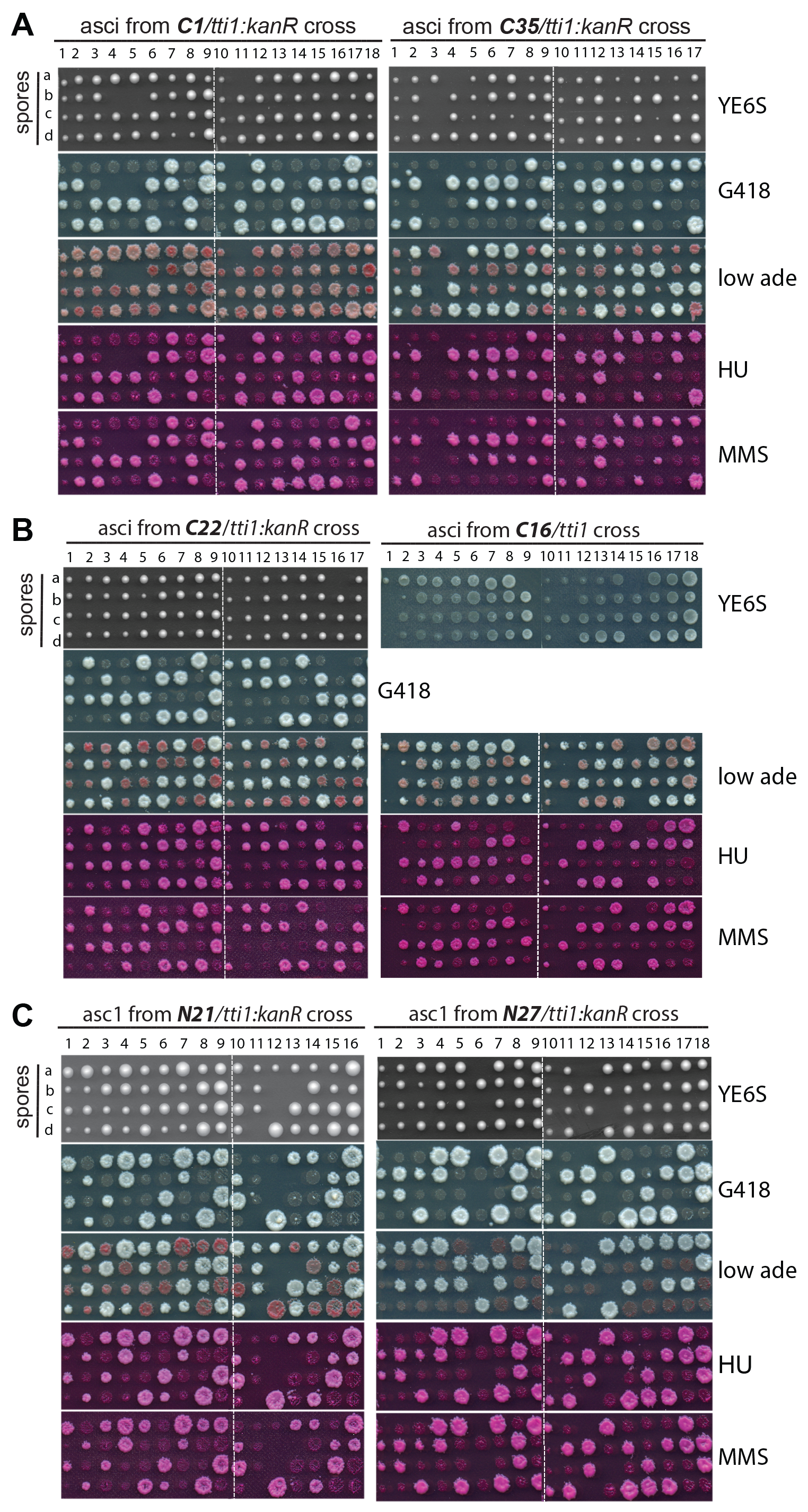

Supplement: S4 Fig — Wild-type S. pombe or a wild-type strain in which tti1 is linked to the kanR marker was crossed with tti1-C1, C35 (A), C22, C16 (B), and N21, N27 (C) mutants. Tetrad dissection was performed for each cross, and the colonies formed on YE6S plates were replica plated on HU and MMS plates containing the lethality dye phloxine B to reveal the mutations, YE6S plates containing G418 to show the kanR marker, and low adenine plates to reveal the two alleles of ade6 as indicated by red or pink colors. All tetrads from each cross showed a 2:2 ratio of kanR, ade6 alleles, and drug sensitive phenotype. The drug phenotype is always segregated from the kanR marker in all crosses, which confirms the successful dissection and the tti1 mutations in all six mutants examined. Tetrad dissection for N18 is shown in Fig 2A. (TIF) [file pgen.1012206.s004.tif]

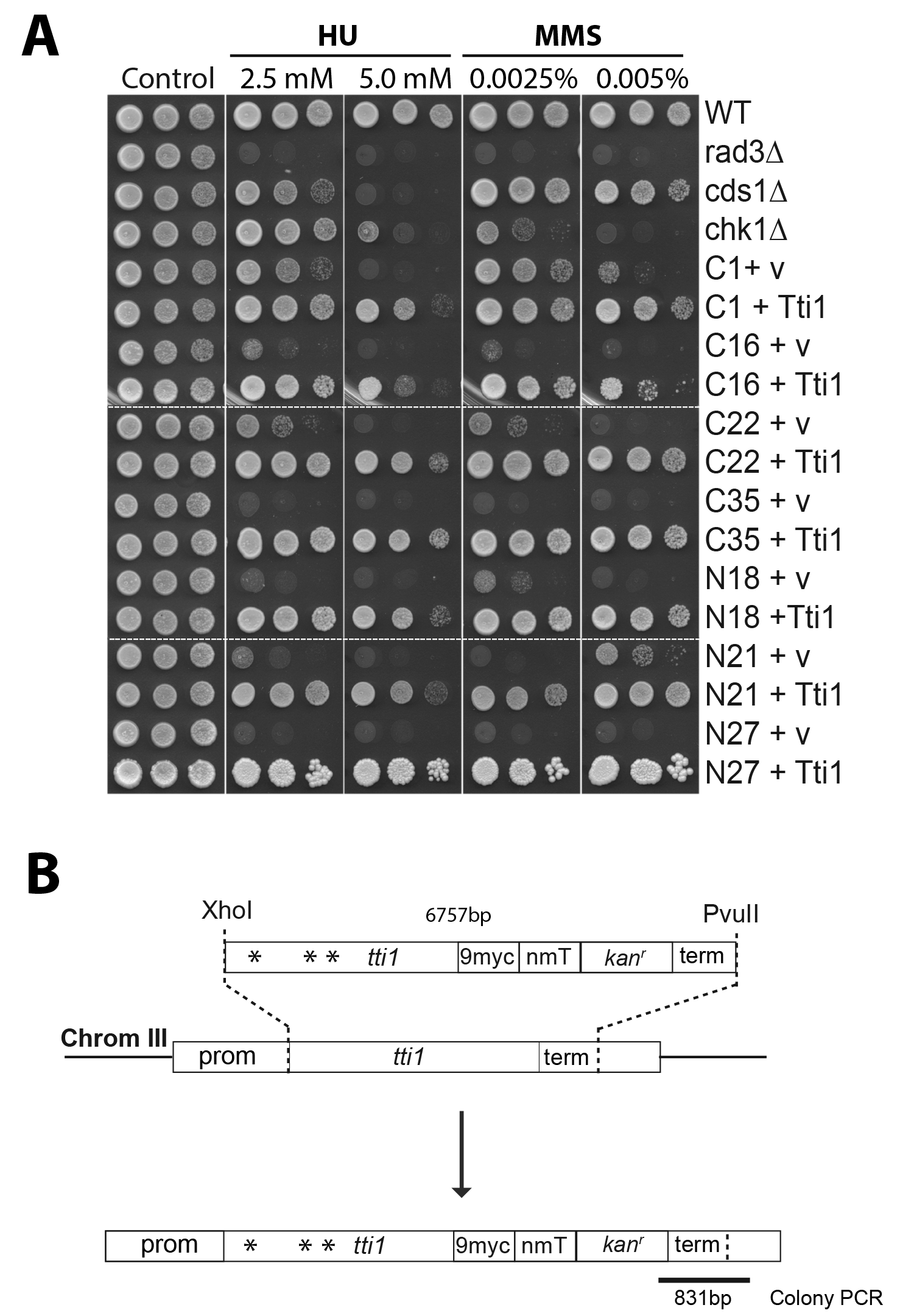

Supplement: S5 Fig — (A) The tti1 mutants were transformed with an empty vector (v) or a vector expressing the wild-type Tti1. Cells were sequentially diluted in ten-fold steps and spotted on YE6S or plates containing HU or MMS at the indicated concentrations. Wild-type S. pombe, rad3∆, cds1∆, and chk1∆ mutants were used as controls. Expression of Tti1 rescued the drug sensitivity and supports the conclusion that all seven tti1 mutants were caused by mutations in tti1. (B) 9myc and nmtT represent the myc epitope tag and nmt1 terminator, respectively. Integrants were screened by colony PCR to ensure successful integration into the genome. Genomic DNA was purified for PCR to confirm integration at the tti1 locus by Sanger sequencing. Western blotting using anti-myc antibody to confirm a protein band of the expected size for Tti1. (TIF) [file pgen.1012206.s005.tif]

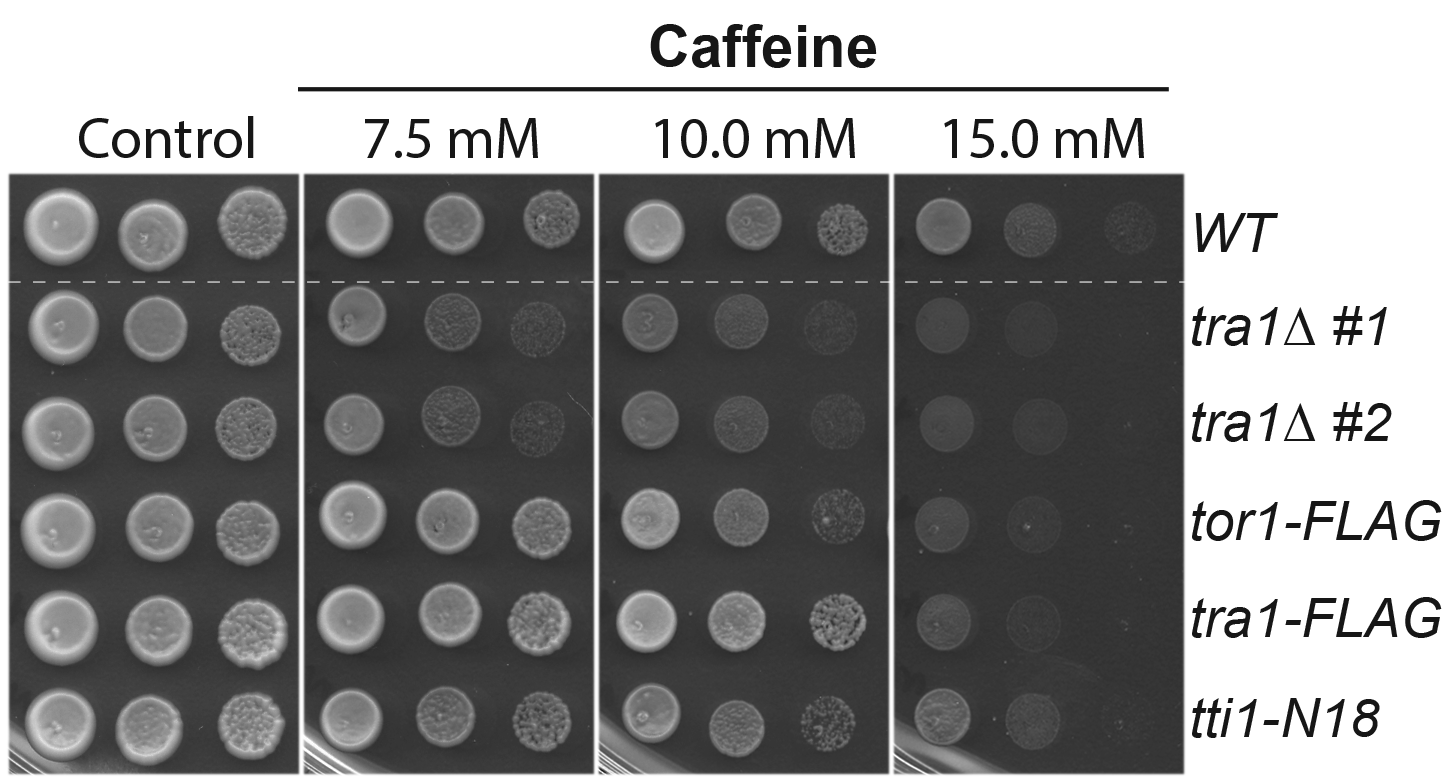

Supplement: S6 Fig — Sensitivities of wild-type S. pombe, tagged strains, and the mutants with the indicated mutations to Caffeine were examined by the three-spot assay. The plates were incubated at 30˚C for three days before being photographed. Two separate colonies of tra1∆ were examined. The dashed line indicates discontinuity. (TIF) [file pgen.1012206.s006.tif]

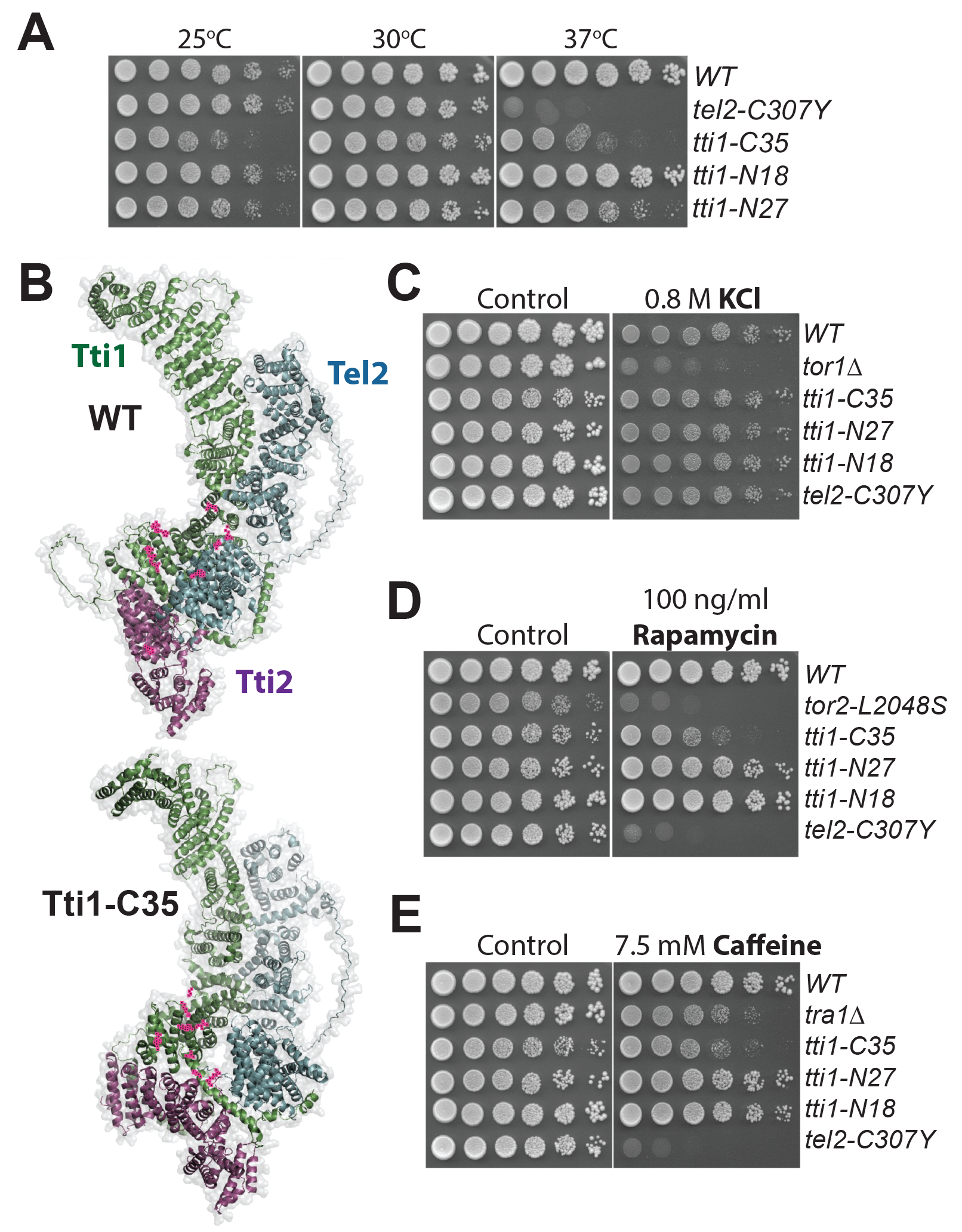

Supplement: S7 Fig — (A) Wild-type S. pombe and the mutants of the indicated mutations were spotted on YE6S and incubated at 25˚C, 30˚C, and 37˚C as in Fig 6D. Unlike the tti1-N18 mutant that grew well under all tested temperatures, the C35 and N27 mutants showed a partial growth defect at 25˚C and 37˚C. (B) The structural impact of the C35 mutation on the TTT complex revealed by AlphaFold3 modeling. The mutated residues were indicated in red in both the wild-type (top) and the mutated TTT complexes (bottom). Comparing with the TTT structure containing Tti1-N18 (Fig 7A), the C35 mutation causes more significant changes to the TTT structure, which is consistent with the broader effect on PIKKs. Spot assay was used to examine the sensitivities of wild-type S. pombe and the indicated mutants to 0.8 M KCl (C), rapamycin (D), and caffeine (E). (TIF) [file pgen.1012206.s007.tif]

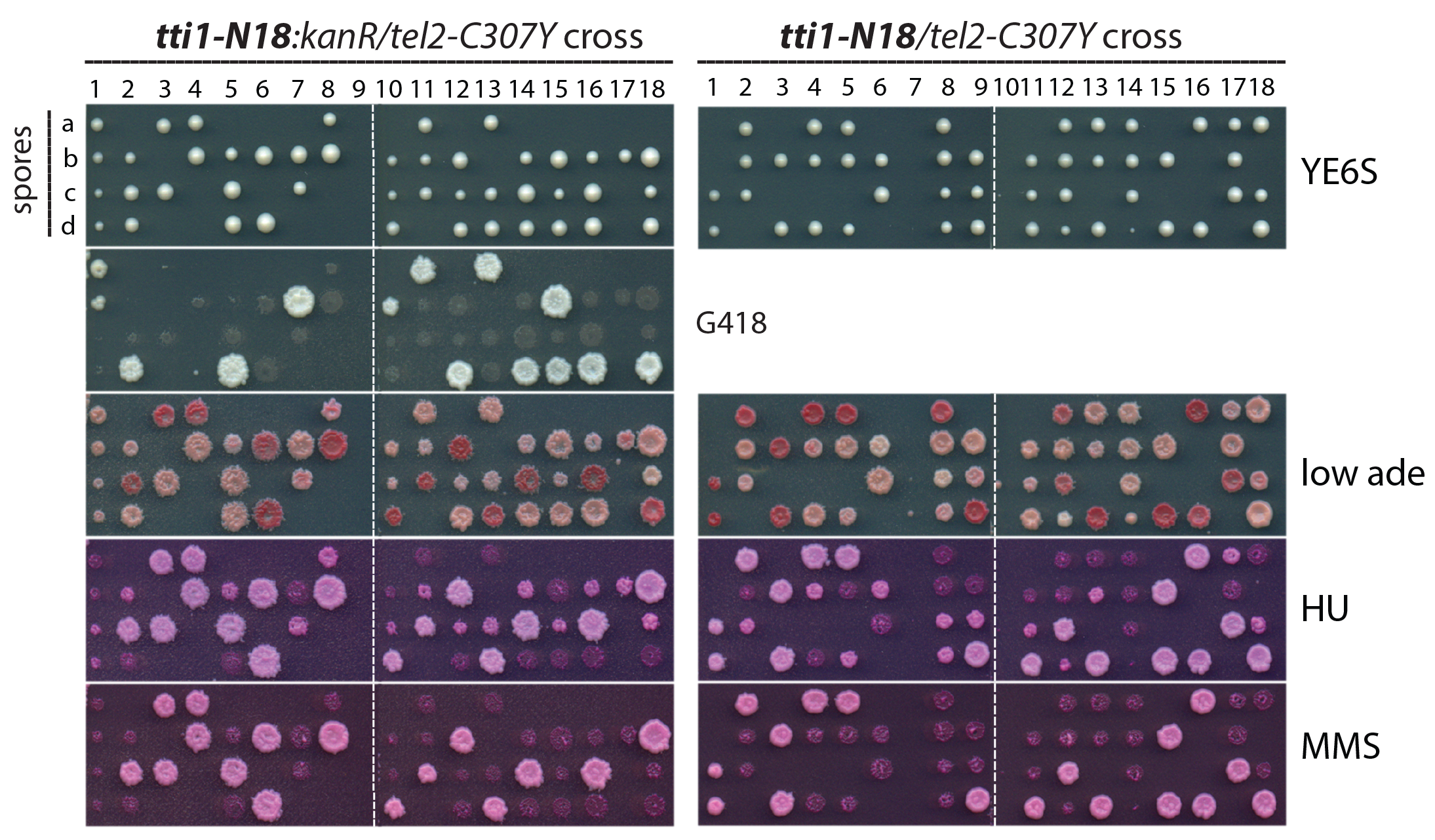

Supplement: S8 Fig — The tti1-N18:kanR integrant and the tti1-N18 primary mutant carrying the ade6-M210 allele were crossed with the tel2-C307Y mutant carrying the ade6-M216 allele. Tetrad dissection was performed on asci from the two crosses. Colonies formed on YE6S plates were replica-plated onto HU and MMS plates containing the lethality dye phloxin B, low adenine plates, and G418 plates to reveal the ade6 alleles and the kanR marker. The dissection results strongly suggest synthetic lethality or severe sickness. (TIF) [file pgen.1012206.s008.tif]
