# Supplementary material for: A tti1 mutation in the Tel2-Tti1-Tti2 complex specifically eliminates the cellular function of Rad3ATR, but not that of other PIKKs in fission yeast
Source: PLoS Genet. 2026 Jun 11;22(6):e1012206. doi: 10.1371/journal.pgen.1012206 (PMC13274921; doi:10.1371/journal.pgen.1012206)
Supplement: S1 Table — (PDF) [file pgen.1012206.s009.pdf]

S1 Table. List of *S. pombe* strains used in this study

Strain	Genotype	Sources
LLD330	h^- ura4-D18	Lab stock
TK2	h^+	Lab stock
TK7	h^- leu1-32 ura4-D18 ade6-M210	Lab stock
TK8	h^+ leu1-32 ura4-D18 ade6-M216	Lab stock
TK48	h^- leu1-32 ade6-M216	Lab stock
NR1826	h^- rad3::ura4 leu1-32 ura4-D18 ade6-M210	Russell lab
TN11	h^+ Δtel1::ura4+ leu1-32 ura4-D18 ade6-M210	Lab stock
YJ1525	h^- Δrad3::ura4+ Δtel1::KanMX 3HA+Rad9(int) leu1-32 ura4-D18 ade6-M210	Lab stock
GBY191	h^+ cds1::ura4 leu1-32 ura4-D18 ade6-M216	Kelly lab
TK197	h^+ Δchk1::ura4+leu1-32 ura4-D18 ade6-M210	Kelly lab
YJ374	h^+ cds1-6his2HA(int) leu1-32 ura4-D18 ade6-M216	Lab stock
LLD3427	h^- chk1-9myc2HA6his:ura4 leu1-32 ura4-D18	Russell lab
YJ1138	h^- 10myc-rad3(int) leu1-32 ura4-D18 ade6-M210	Lab stock
YJ1574	h^+ 10myc-rad3(int) leu1-32 ura4-D18 ade6-M216	Lab stock
YJ1576	h^+ 9myc:tel1(int) leu1-32 ura4-D18 ade6-M216	Lab stock
YJ1481	h^+ tel2-C307Y	Lab stock
YJ1475	h^+ tel2-C307Y cds1-6his2HA(int) leu1-32 ura4-D18 ade6-M210	Lab stock
YJ1515	h^+ tel2-C307Y-3HA:ura4 leu1-32 ade6-M210	Lab stock
YJ1520	h^+ tel2(C307Y) chk1-9myc-2HA6his:ura4+ leu1-32 ura4-D18	Lab stock
YJ1536	h^+ 10Myc-Rad3(int) tel2(C307Y)leu1-32 ura4-D18 ade6-M210	Lab stock
YJ1544	h^- 9myc:tel1(int) tel2(C307Y) leu1-32 ura4-D18	Lab stock
SK6	h^- tti1-9myc-nmtTERM-kanR(int) leu1-32 ura4-D18 ade6-M210	Lab stock
SK7	h^+ tti1-9Myc-nmtT:KanMX6(int) tel2(WT)-3HA-nmtT-ura4+(int) leu1-32 ura4-D18 ade6-M210	Lab stock
FY21023	h^- tel2-3HA:ura4+ leu1-32 ura4-D18	Lab stock
YJ1548	h^- 7myc-tti2-nmtTERM-kanR-c(int) leu1-32 ura4-D18 ade6-M210	Lab stock
YJ1549	h^- 7myc-tti2-nmtTERM-kanR-c(int) tel2-3HA-nmtT:ura4	Lab stock
TA390	h^- tor1Δ::ura4+ ura4-D18	Lab stock
FY21019	h^- tor2 ts[L2048S][kanR]	YGRC
FY32131	h? tra1::kanR lid1-6 leu1-32 ura4-D18 ade6-M21X	YGRC
DHP682	h^- hphMX6+::FLAG3-tor1	Helmlinger Lab
DHP680	h^- kanMX6+::FLAG3-tor2	Helmlinger Lab
DHP1512	h^- FLAG3-tra1	Helmlinger Lab
FY21012	h^+ FLAG-tor2:kanR	Lab stock
SB2733	h^+ tti1-C1 cds1-6his2HA(int) leu1-32 ura4-D18 ade6-M216	This study
SB2734	h^+ tti1-C16 cds1-6his2HA(int) leu1-32 ura4-D18 ade6-M216	This study
SB2735	h? tti1-C22 cds1-6his2HA(int) leu1-32 ura4-D18 ade6	This study
SB2736	h? tti1-C35 cds1-6his2HA(int) ura4-D18 ade6-M210	This study
SB2737	h^+ tti1-N18[P70Q-I367V-G457D] cds1-6his2HA(int) leu1-32 ura4-D18 ade6	This study
SB2738	h^- tti1-N21 cds1-6his2HA(int) ura4-D18 ade6-M210	This study
SB2740	h^- tti1-N27 cds1-6his2HA(int) ura4-D18 ade6-M216	This study
SB2741	h^- tti1-C1 chk1-9myc2HA6his:ura4 ade6	This study
SB2742	h^+ tti1-C16 chk1-9myc2HA6his:ura4 leu1-32 ade6-M210	This study
SB2743	h^- tti1-C22 chk1-9myc2HA6his:ura4 ade6	This study
SB2744	h^+ tti1-C35 chk1-9myc2HA6his:ura4 leu1-32 ade6	This study
SB2745	h^+ tti1-N18[P70Q-I367V-G457D] chk1-9myc2HA6his:ura4 leu1-32 ade6	This study

SB2746	*h+ tti1-N21 chk1-9myc2HA6his:ura4 leu1-32 ade6*	This study
SB2748	*h? tti1-N27 chk1-9myc2HA6his:ura4 ade6*	This study
SB2749	*h+ tti1-C1 10myc-rad3(int) leu1-32 ura4-D18 ade6-M216*	This study
SB2750	*h- tti1-C16 10myc-rad3(int) leu1-32 ura4-D18 ade6-M210*	This study
SB2751	*h+ tti1-C22 10myc-rad3(int) leu1-32 ura4-D18 ade6-M216*	This study
SB2752	*h+ tti1-C35 10myc-rad3(int) leu1-32 ura4-D18 ade6*	This study
SB2753	*h- tti1-N18[P70Q-I367V-G457D] 10myc-rad3(int) ura4-D18 ade6-M216*	This study
SB2754	*h- tti1-N21 10myc-rad3(int) ura4-D18 ade6-M216*	This study
SB2756	*h- tti1-N27 10myc-rad3(int) leu1-32 ura4-D18 ade6-M210*	This study
SB2758	*h+ tti1-N18[P70Q-I367V-G457D] leu1-32 ura4-D18 ade6-M216*	This study
SB2781	*h- tti1-N18[P70Q-I367V-G457D] leu1-32 ura4-D18 ade6-M216*	This study
SB2759	*h+ tti1-N18[P70Q-I367V-G457D] rad3::ura4 leu1-32 ade6-M216*	This study
SB2760	*h- tti1-N18[P70Q-I367V-G457D] tel1::ura4 ade6-M210*	This study
YJ2853	*h+ tti1-N18[P70Q-I367V-G457D]9myc-kanR(int) leu1-32 ura4-D18 ade6-M216*	This study
SB2761	*h+ tti1-N18[P70Q-I367V-G457D]9myc-kanR(int) tel2-3HA:ura4+ leu1-32 ade6-M216*	This study
SB2782	*h- tti1-N18[P70Q-I367V-G457D] 9myc:tel1(int) ura4-D18 ade6-M216*	This study
SB2785	*h- tti1-N18[P70Q-I367V-G457D] hphMX6+::FLAG3-tor1 ade6*	This study
SB2786	*h- tti1-N18[P70Q-I367V-G457D] FLAG-tor2:kanR leu1-32 ade6*	This study
SB2854	*h- tti1-N18[P70Q-I367V-G457D] FLAG3-tra1 ade6-M216*	This study
SB2857	*h- tti1-N18[P70Q-I367V-G457D] 7myc-tti2-nmtTERM-kanR-c(int) tel2-3HA-nmtT:ura4 leu1-32 ade6-M210*	This study
