# Supplementary material for: A tti1 mutation in the Tel2-Tti1-Tti2 complex specifically eliminates the cellular function of Rad3ATR, but not that of other PIKKs in fission yeast
Source: PLoS Genet. 2026 Jun 11;22(6):e1012206. doi: 10.1371/journal.pgen.1012206 (PMC13274921; doi:10.1371/journal.pgen.1012206)
Supplement: S2 Table — (PDF) [file pgen.1012206.s010.pdf]

S2 Table. List of plasmids used in this study.

Name	Description	Sources
pYJ1821	*pJK210+prom-tti1(NdeI-NheI-XmaI)-term/ura4+*	This study
pYJ2205	*pGEM7-Tti1(XhoI-NheI-NotI P70Q-I367V-G457D)9myc-nmtTERM-Kan-Tti1C-term*	This study
pSK12	*pGEM+Tti1-9myc-nmtTERM-KanMX-Tti1(T)*	This study
pSK38	*pIRT-2U+prom+Tti1+term(PstI-Sa1I)*	This study
