# Supplementary material for: A tti1 mutation in the Tel2-Tti1-Tti2 complex specifically eliminates the cellular function of Rad3ATR, but not that of other PIKKs in fission yeast
Source: PLoS Genet. 2026 Jun 11;22(6):e1012206. doi: 10.1371/journal.pgen.1012206 (PMC13274921; doi:10.1371/journal.pgen.1012206)
Supplement: S3 Table — (PDF) [file pgen.1012206.s011.pdf]

S3 Table. List of PCR and sequencing primers used in this study.

**Name**	**Sequence** (5´ -> 3´)	**Note**
Tti1(P)(XhoI)f	ttatCtCgagcaattttcatatctgtc	Cloning
Tti1(XhoI)f	gtttctcgagATGTCTCATATACAGAGTAT	Cloning & Colony PCR
Tti1(NotI)b	tttaGCGGCCGCTAATGATGGAAATCAATGGTTTT	Cloning & Colony PCR
Tti1(T)(NotI)b	ctaaGcGGCcgctcatccaaacgtattg	Cloning
Tti1(XmaI)b	tttaCCCGGgTTAAATGATGGAAATCAATG	Cloning
Tti1(P)f	CTGGGAATTGGCTGTGCATCTAG	Sequencing
Tti1(480-505)f	ACAGTATCTTCTCTTTGCGAGAGCTC	Sequencing
Tti1(662-641)b	GTCAATCCCGATACTATGCCAG	Sequencing
Tti1(1048-69)f	AGGGACTATGGGATGAACAAAC	Sequencing
Tti1(1633-54)f	GTCTTTACGAAAGCATCGTTGG	Sequencing
Tti1(1692-15)f	TTTGTGTGTTCGTTCTTGCATTGC	Sequencing
Tti1(2165-88)f	TGGAGGAGGACGCAATGAACGAGG	Sequencing & Colony PCR
Tti1(2844-69)f	AAGTAACTTCAGCATGTGTGGCTAAC	Sequencing & Colony PCR
Tti1(1811-88)b	GAAGCAAACGCCAAATGTTCAAGG	Sequencing
Tti1(T)(SalI)f	TTCTgTcGACAATTTGTTACACTTGAA	Cloning
Tti1(T)(BglII)b	ggtaAgAtcttccaggcagaccaaatc	Cloning
