# Supplementary material for: A tti1 mutation in the Tel2-Tti1-Tti2 complex specifically eliminates the cellular function of Rad3ATR, but not that of other PIKKs in fission yeast
Source: PLoS Genet. 2026 Jun 11;22(6):e1012206. doi: 10.1371/journal.pgen.1012206 (PMC13274921; doi:10.1371/journal.pgen.1012206)
Supplement: Attachment — Submitted filename: Response to Reviewers comments.pdf [file pgen.1012206.s014.pdf]

Response to reviewers' comments.
Note: reviewers' comments are in **black**, and the responses are **blue.**

Reviewer #1: General Comment
The current model proposes that the Tel2–Tti1–Tti2 (TTT) complex regulates the maturation of all PIKKs. The authors have identified and characterized an interesting separation-of-function mutation, tti1-N18, and propose that this allele is specifically defective in Rad3 kinase function. However, given the broad role of TTT in PIKK regulation, additional evidence is required to demonstrate that the phenotypes observed in tti1-N18 mutants are uniquely attributable to compromised Rad3 kinase activity rather than to more general defects in PIKK stability, maturation, or signaling. The tti1-N18 mutation could also influence other components within the Rad3 pathway or affect parallel PIKK-dependent processes. Overall, while the proposed model is intriguing, further experimental validation is necessary to establish that this mutation specifically impairs Rad3 function rather than broadly affecting PIKK regulation.

1. Potential involvement of other PIKKs
Because the Tel2–Tti1–Tti2 (TTT) complex promotes the maturation of all PIKKs, it remains possible that the tti1-N18mutation affects additional PIKKs beyond Rad3. The assay presented in Fig. 6 may not be sufficiently sensitive to detect subtle or partial defects in other PIKK pathways. A more comprehensive analysis of multiple PIKK-dependent outputs using different read-outs—such as kinase activity, substrate phosphorylation—would strengthen the claim that tti1-N18 is specifically defective in Rad3 function.

Response: We greatly appreciate the reviewers' time and effort in reviewing the manuscript. Regarding the comments and suggestions from the 1st reviewer, we generally agree with them all. Besides Rad3 and Tel1, the remaining four PIKKs in fission yeast are Tor1, Tor2, Tra1, and Tra2. Since Tor2 and Tra2 are essential for cell growth, and there is no growth defect observed in *tti1-N18* at 25˚C, 30˚C, and 37˚C, the functions of Tor2 and Tra2 are largely uncompromised. Consistent with a functional Tor2, *tti1-N18* is found resistant to Rapamycin and Torin like wild type cells, unlike the *tor2-L2048S* mutant (Fig 6). Furthermore, while a wild-type caffeine resistance was observed in *tti1-N18* in Fig 6, confirming the functional Tra1, the wild-type resistance to 0.8 M KCl is consistent with the functional Tor1 (Fig 6). In addition to these results, the dual Tor-dependent switch: Tor2 inactivation and essential Tor1 activation drives G1 cell cycle arrest, which is required for proper mating. As shown in Fig 2A, we have observed efficient mating activity in *tti1-N18*. Together, these observations support our conclusion that the functions of the remaining four PIKKs are largely unaffected in the *tti1-N18* mutant, although we cannot rule out the possibility that they are minimally or mildly compromised.

The reviewer suggested other readout assays, such as kinase activity or substrate phosphorylation. Although they are good suggestions that may provide more evidence, they do not apply to Tra1 and Tra2, because they are pseudokinases that lack kinase activity (PMID: 21642955). The established readout for Tor2 and Tor1 kinases is phosphorylation of Psk1, the S. pombe ortholog of S6 Kinase/p70S6K, and Gad8, the fission yeast AKT/SGK. Unfortunately, phosphor-specific antibodies for these substrates are not available in fission yeast, which prevents us from examining the kinase activities of Tor1 and Tor2 by monitoring substrate phosphorylation, like the phosphorylation of Mrc1, Cds1, and Chk1 by Rad3 and Tel1, shown in Fig 1D to F, 4A and B. It would be interesting to study these phosphorylation events by Tor1 and Tor2 in the future, which, in our opinion, is beyond the scope of this paper at this stage.

2. Because TTT appears to be destabilized in the tti1-N18 mutant (Fig. 7), it is unclear why the resulting phenotype would be specific to the Rad3 pathway. If TTT function is globally compromised, one might expect defects across multiple PIKK pathways.

One possible explanation is that different PIKKs may vary in their expression levels and turnover rates. For example, if Rad3 is expressed at higher levels for full function or has a faster turnover rate than other PIKKs, it may be more sensitive to partial TTT dysfunction, thereby producing an apparently Rad3-specific phenotype.

Response: We appreciate the suggestions and have added these possibilities in the Discussion (see lines 508-511).

Reviewer 2

The Tel2–Tti1–Tti2 (TTT) complex functions as a chaperone for the translational maturation of all PIKKs, including the checkpoint kinases Rad3^ATR and Tel1^ATM in *S. pombe*. In this study, the authors identified seven *tti1* mutants that are sensitive to genotoxic stress through PCR-based mutagenesis. Notably, several of these mutants exhibit selective defects in Rad3^{ATR} function, suggesting that specific perturbations in Rad3 maturation can occur independently of other PIKKs. This work successfully uncovers substrate-specific TTT mutants, which will be valuable for elucidating the mechanistic role of the TTT complex in PIKK maturation; although additional experiments are needed to clarify the precise defects associated with these newly identified *tti1* alleles. The manuscript should be suitable for publication after the following concerns are addressed.

Major Points

- **Figure 7 – Reduced TTT complex formation in *tti1-N18*.**

The authors report decreased TTT complex assembly in the *tti1-N18* mutant. As they acknowledge in the Discussion, such a reduction should compromise the stability of all PIKKs, which contradicts the selective phenotype of *tti-N18* observed in this study. Therefore, the reduced complex formation is unlikely to account for the specific defects attributed to *tti1-N18*. Moving this figure to the supplemental materials may be more appropriate.

Response: We appreciate the suggestion. Exactly how TTT controls the functions and protein stability of PIKKs remains to be fully understood. We agree with the reviewer that partially destabilized TTT should have a broader effect on PIKKs. However, destabilization is apparently not the only explanation, as we showed that although tel2-C307Y, tti1-C35, and tti1-N18 all destabilize the TTT, the effects of TTT on PIKKs for the three mutants are, however, quite different. Although it is somewhat inconsistent with the current mechanism, we prefer Fig 7 as the main figure, because it raises interesting and important questions for future studies.

- **Figure 4 – Distinguishing Rad3 vs. Tel1 function in *tti1-N18*.**

The data suggest that *tti1-N18* abolishes Rad3-dependent phosphorylation of Mrc1 at T645 while leaving Tel1-dependent Mrc1 mobility shift intact. To strengthen this conclusion, the authors should compare the genotoxin sensitivity of *tti1-N18*, *tel1Δ* single, and *tti1-N18 tel1Δ* double mutants, especially given the synthetic DNA damage sensitivity previously reported for *rad3Δ tel1Δ* strains.

Response: We appreciate the suggestions and performed the spot assay. While the tel1Δ single mutant shows wild-type resistance to HU, as Rad3 is functional, the double mutants of *tti1-N18 tel1Δ, tti1-N18 rad3Δ,* and *tel1Δ rad3Δ* showed the HU sensitivity as in rad3Δ cells (Fig 4C).

- **Figure 5 – PIKK protein level analyses in *tti1-N18* mutants.**

A key conclusion is that *tti1-N18* reduces Rad3 protein levels without affecting other PIKKs. While Tel1, Tor1, and Tor2 levels were measured in three biological replicates, additional replicates assessing Rad3 levels are needed to robustly support this claim.

Response: Following the suggestion, we repeated the Western blotting analysis in triplicate samples, which confirms the moderate reduction of Rad3 protein level in *tti1-N18* (Fig 5A). The original Fig 5A is moved to Fig 1 as 1G.

Minor Points

- **Figure 1E – Cds1 T11 phosphorylation.**

The phosphorylation of Cds1 at T11 by Rad3 appears absent in all seven *tti1* mutants, which does not align with the differences observed in Mrc1 T645 phosphorylation. A longer exposure of the Cds1 blot may give more information regarding these discrepancies.

Response: Following the suggestion, we added a longer exposure of the blot. Indeed, the weak signals become more visible upon longer exposure (Fig 1E, middle panel).

- **Figure 1F – Chk1 phosphorylation vs. MMS sensitivity.**

The *tti1-N27* and *tti1-C35* mutants display strong MMS sensitivity, comparable to *chk1Δ*. However, the quantification of Chk1 phosphorylation in these mutants does not reflect this phenotype. This inconsistency should be addressed.

Response: Quantification of Chk1 phosphorylation is always challenging due to the subtle shift. Comparing with untreated wt cells (Fig 1F, leftmost lane), Chk1 phosphorylation in tti1-N27 and tti1-C35 is at or below the background level.

- **Figure 2 – Cell length phenotype of *tti1-C22*.**

Although *tti1-C22* shows fewer abnormal mitotic cells, the mutant cells are clearly shorter than wild-type following HU treatment. This observation should be discussed.

Response: We appreciate the suggestions and added the possible explanation to the revised manuscript (see lines 294-296). The tti1-C22 behaves like the metabolic mutants we identified before that undergo oxidative stress induced by HU.

- **Figure S4 – Tetrad dissection verification.**

The verification of *tti1* mutants via tetrad analysis should be presented earlier in the Results section, prior to phenotype characterization.

Response: We appreciate the suggestion and have reordered figures 2 and 3, which streamlines the information flow logically and enhances the readability.

- **Temperature-sensitive mutants (*tti1-C35* and *tti1-N27*).**

Given their temperature-sensitive phenotypes, it would be informative to assess whether these mutants are also sensitive to KCl, rapamycin, or caffeine.

Response: We performed the suggested experiment to further evaluate the sensitivity of tti1-C35 and tti1-N27 to KCl, rapamycin, and caffeine (S6 C-E). The results show that *tti1-C35* is sensitive to

rapamycin and caffeine but not sensitive to KCl, suggesting defective Tor2 and Tra1, not Tor1. In contrast, *tti1-N27* is resistant to all three agents tested, resembling the behavior of *tti1-N18*. These findings indicate that, in the temperature-sensitive *tti-C35* mutant, besides the defective Rad3 and Tel1, Tor2, Tra1, and Tra2 are partially impaired, suggesting a much broader effect on PIKKs. Conversely, Tel1, Tor1, Tor2, Tra1, and Tra2 remain functional in tti1-N27, except for the slower growth at 25˚C and 37˚C, suggesting that the essential PIKKs Tor2 or Tra2 may remain functional but mildly affected. On the contrary, the tti1-N18 shows wild-type growth at all temperatures tested.

- **Line 308 – Terminology.**

Describing slow growth at 25 °C as "cold-sensitive" is not appropriate and should be revised.

Response: Revised.
