# Supplementary material for: A tti1 mutation in the Tel2-Tti1-Tti2 complex specifically eliminates the cellular function of Rad3ATR, but not that of other PIKKs in fission yeast
Source: PLoS Genet. 2026 Jun 11;22(6):e1012206. doi: 10.1371/journal.pgen.1012206 (PMC13274921; doi:10.1371/journal.pgen.1012206)
Supplement: Attachment — Submitted filename: Response to Reviewers comments[2].pdf [file pgen.1012206.s015.pdf]

**Response to reviewer #1's comments.**

Note: Reviewer #1's comments are in **black**, and the responses are **blue.**

Reviewer #1: I agree that the tti1-N18 mutation exhibits a much stronger defect in the Rad3 pathway compared to other PIKK pathways, and this phenotype is interesting. However, the revision does not provide a satisfactory explanation for this difference.

The authors argue that, because Tra1 and Tra2 are not kinases, these pathways cannot be analyzed in the same way; however, this point is not fully convincing. Tra1 and Tra2 have well-established roles in transcription, and transcriptional outputs can therefore serve as functional readouts for their activity. Similarly, Tor1 and Tor2 signaling regulates transcriptional programs, including nutrient-responsive gene expression and ribosome biogenesis. Thus, comparable transcription-based analyses could, in principle, be used to assess defects in these pathways as well. This issue is not addressed in the revision.

In addition, a more careful analysis using higher concentrations of stress-inducing agents would strengthen the conclusions. Notably, even in the cell proliferation assays, the tti1-18 mutant forms smaller colonies in the presence of caffeine, suggesting a measurable growth defect that warrants further investigation (Fig. 6).

**Response:** We appreciate the careful review provided by reviewer #1 and apologize for our misunderstanding of the suggestion. We agree with the reviewer that more mechanistic studies, such as transcription-based analyses, would enhance our results on four other PIKKs: Tra1, Tra2, Tor1, and Tor2. These studies should be carried out in the future for a better understanding of the TTT complex (see lines 517-519 in Discussion). However, the experiments, in our opinion, are beyond the scope of the current study.

Regarding the results in Fig 6, we appreciate the suggestion and have changed the description of the caffeine sensitivity of the *tti1-N18* mutant as suggested (see lines 388-392 on page 18). We also added our results from months ago on the inhibitory effect on cell growth by caffeine using concentrations ranging from 7.5 mM to 15.0 mM (S6 Fig in the revised manuscript). The results clearly showed that, consistent with the results shown in Fig 6C, caffeine slightly suppressed cell growth of the tti1-N18 mutant (as well as the tagged strains of tor1 and tra1) even at 15 mM, as compared with wild-type *S. pombe* under similar conditions.
