# Supplementary material for: A tti1 mutation in the Tel2-Tti1-Tti2 complex specifically eliminates the cellular function of Rad3ATR, but not that of other PIKKs in fission yeast
Source: PLoS Genet. 2026 Jun 11;22(6):e1012206. doi: 10.1371/journal.pgen.1012206 (PMC13274921; doi:10.1371/journal.pgen.1012206)
Supplement: Attachment — Submitted filename: PloSG-2026-a.docx [file pgen.1012206.s012.docx]

**PloS-Genetics 2026**

The Tel2–Tti1–Tti2 (TTT) complex functions as a chaperone for the translational maturation of all PIKKs, including the checkpoint kinases Rad3^ATR and Tel1^ATM in *S. pombe*. In this study, the authors identified seven *tti1* mutants that are sensitive to genotoxic stress through PCR‑based mutagenesis. Notably, several of these mutants exhibit selective defects in Rad3^ATR^ function, suggesting that specific perturbations in Rad3 maturation can occur independently of other PIKKs. This work successfully uncovers substrate‑specific TTT mutants, which will be valuable for elucidating the mechanistic role of the TTT complex in PIKK maturation; although additional experiments are needed to clarify the precise defects associated with these newly identified *tti1* alleles. The manuscript should be suitable for publication after the following concerns are addressed.

**Major Points**

- **Figure 7 – Reduced TTT complex formation in *tti1‑N18*.**
  The authors report decreased TTT complex assembly in the *tti1‑N18* mutant. As they acknowledge in the Discussion, such a reduction should compromise the stability of all PIKKs, which contradicts the selective phenotype of *tti-N18* observed in this study. Therefore, the reduced complex formation is unlikely to account for the specific defects attributed to *tti1‑N18*. Moving this figure to the supplemental materials may be more appropriate.
- **Figure 4 – Distinguishing Rad3 vs. Tel1 function in *tti1‑N18*.**
  The data suggest that *tti1‑N18* abolishes Rad3‑dependent phosphorylation of Mrc1 at T645 while leaving Tel1‑dependent Mrc1 mobility shift intact. To strengthen this conclusion, the authors should compare the genotoxin sensitivity of *tti1‑N18*, *tel1Δ* single, and *tti1‑N18 tel1Δ* double mutants, especially given the synthetic DNA damage sensitivity previously reported for *rad3Δ tel1Δ* strains.
- **Figure 5 – PIKK protein level analyses in *tti1-N18* mutants.**
  A key conclusion is that *tti1‑N18* reduces Rad3 protein levels without affecting other PIKKs. While Tel1, Tor1, and Tor2 levels were measured in three biological replicates, additional replicates assessing Rad3 levels are needed to robustly support this claim.

**Minor Points**

- **Figure 1E – Cds1 T11 phosphorylation.**
  The phosphorylation of Cds1 at T11 by Rad3 appears absent in all seven *tti1* mutants, which does not align with the differences observed in Mrc1 T645 phosphorylation. A longer exposure of the Cds1 blot may give more information regarding these discrepancies.
- **Figure 1F – Chk1 phosphorylation vs. MMS sensitivity.**
  The *tti1‑N27* and *tti1‑C35* mutants display strong MMS sensitivity, comparable to *chk1Δ*. However, the quantification of Chk1 phosphorylation in these mutants does not reflect this phenotype. This inconsistency should be addressed.
- **Figure 2 – Cell length phenotype of *tti1‑C22*.**
  Although *tti1‑C22* shows fewer abnormal mitotic cells, the mutant cells are clearly shorter than wild‑type following HU treatment. This observation should be discussed.
- **Figure S4 – Tetrad dissection verification.**
  The verification of *tti1* mutants via tetrad analysis should be presented earlier in the Results section, prior to phenotype characterization.
- **Temperature‑sensitive mutants (*tti1‑C35* and *tti1‑N27*).**
  Given their temperature‑sensitive phenotypes, it would be informative to assess whether these mutants are also sensitive to KCl, rapamycin, or caffeine.
- **Line 308 – Terminology.**
  Describing slow growth at 25 °C as “cold‑sensitive” is not appropriate and should be revised.
